# The current understanding of the phenotypic and functional properties of human regulatory B cells (Bregs)

**DOI:** 10.1093/oxfimm/iqae012

**Published:** 2024-09-20

**Authors:** Nawara Faiza Ahsan, Stella Lourenço, Dimitra Psyllou, Alexander Long, Sushma Shankar, Rachael Bashford-Rogers

**Affiliations:** Department of Biochemistry, University of Oxford, Oxford OX1 3QU, United Kingdom; Centre for Human Genetics, University of Oxford, Oxford OX3 7BN, United Kingdom; Keizo Asami Institute, Federal University of Pernambuco, Recife 50740-520, Brazil; Department of Biochemistry, University of Oxford, Oxford OX1 3QU, United Kingdom; Department of Biochemistry, University of Oxford, Oxford OX1 3QU, United Kingdom; Nuffield Department of Surgical Sciences, University of Oxford, Oxford OX3 7DQ, United Kingdom; Department of Biochemistry, University of Oxford, Oxford OX1 3QU, United Kingdom; Oxford Cancer Centre, University of Oxford, Oxford OX3 7LH, United Kingdom

**Keywords:** B cells, regulation, Bregs, autoimmune disease, infection, IL-10, IL-25, TGF-B

## Abstract

B cells can have a wide range of pro- and anti- inflammatory functions. A subset of B cells called regulatory B cells (Bregs) can potently suppress immune responses. Bregs have been shown to maintain immune homeostasis and modulate inflammatory responses. Bregs are an exciting cellular target across a range of diseases, including Breg induction in autoimmunity, allergy and transplantation, and Breg suppression in cancers and infection. Bregs exhibit a remarkable phenotypic heterogeneity, rendering their unequivocal identification a challenging task. The lack of a universally accepted and exclusive surface marker set for Bregs across various studies contributes to inconsistencies in their categorization. This review paper presents a comprehensive overview of the current understanding of the phenotypic and functional properties of human Bregs while addressing the persisting ambiguities and discrepancies in their characterization. Finally, the paper examines the promising therapeutic opportunities presented by Bregs as their immunomodulatory capacities have gained attention in the context of autoimmune diseases, allergic conditions, and cancer. We explore the exciting potential in harnessing Bregs as potential therapeutic agents and the avenues that remain open for the development of Breg-based treatment strategies.

## Introduction

B cells are key players in shaping the humoral immune system by antibody production, opsonization, antigen presentation, and activation of T cells. B cells are historically acknowledged as promoters of adaptive immune responses. An imbalance or dysfunction of B cells can result in suboptimal defence against infections, impaired cancer surveillance, and a breakdown of immune tolerance causing autoimmune or autoinflammatory diseases. More recent studies have recognized the immunoregulatory properties of B cells including maintenance of immune tolerance, and suppression of immune responses. In particular, a subset of B cells named regulatory B cells (Bregs) have demonstrated immune suppressive properties in conditions such as allergies, autoimmune diseases, infection, transplantation, and tumours.

The presence of a B cell population with immunosuppressive properties was first suggested in the 1970s, based on findings that B cell-depleted splenocytes correlated with the severity and duration of delayed-type hypersensitivity (DTH) in guinea pig models [1, 2]. This hypothesis of a suppressive B cell population was further supported by studies demonstrating that B cells regulated the differentiation of T cells into suppressor T cells and could act as a suppressive-inducer in feedback control [2, 3]. Janeway *et al.* confirmed the existence of an immunoregulatory subset of B cells which when adoptively transferred in B cell-deficient mice enabled complete recovery from experimental autoimmune encephalomyelitis (EAE) [3]. Soon after, Mizoguchi and colleagues reported a suppressive function of B cells in colitis and intestinal inflammation in mice models and named them ‘regulatory B cells’ [4–6]. Menna Clatworthy’s team were the first to report findings that suggested the existence of human Bregs when they observed that the use of an anti-CD20 monoclonal antibody therapy that depleted B cells in transplant recipients increased the rate of organ rejection, such that the clinical trial was ceased [7]. Human Bregs were then more firmly identified in 2010 by Claudia Mauri’s group in the context of Systemic Lupus Erythematosus (SLE) [8]. Since then, our understanding of the characteristics, phenotypes, and functionalities of Bregs in the context of cancer, infection, and other autoimmune diseases has significantly increased.

Briefly, Bregs are capable of modulating immune responses by inhibiting effector functions of CD4^+^ and CD8^+^ T cells, promoting differentiation of regulatory T cells (Tregs), and producing immunosuppressive cytokines such as Interleukin 10 (IL-10), Transforming growth factor beta (TGF-β), Interleukin 35 (IL-35) [9]. Further, these cells can suppress monocyte proliferation, and interferon-gamma (IFN-γ) production by natural killer (NK) cells, in an IL-10 dependent manner [10]. While Breg deficiencies and/or defects have been seen in autoimmune disorders [6], chronic inflammation [11], and graft rejection [12], their abundance has been associated with tumour progression and poorer outcomes in cancer [12], in both animal models and humans. Such diverse functional properties of Bregs makes them potential cellular targets across a range of diseases, including Breg suppression or depletion in cancers and infection, or Breg induction in autoimmunity, allergy, and transplantation. However, harnessing the clinical potential of Bregs is limited by our poor understanding of human Breg biology.

While our insights on the biology of Bregs have expanded in recent decades, most research has been conducted on murine Bregs. As such our understanding of the origin and differentiation of human Bregs remains unclear, which is further hampered by a lack of unique phenotypic markers or transcriptional markers for characterizing human Bregs, as well as context-dependant differences in phenotypes and secretory products and discrepancies amongst papers causing further complications. This review discusses our current knowledge on origin, differentiation, and phenotypic diversity of human Bregs, and elaborates on the functional roles of Bregs in cancer, infection, and autoimmune diseases highlighting opportunities for clinical application and future research.

## Origin and differentiation of Bregs

The progenitors of B cells differentiate from common lymphoid progenitors in the bone marrow, where they undergo a series of progressively committed differentiation steps into early pro-B, pro-B, and pre-B cells. Pre-B cells develop into immature B cells that leave the bone marrow becoming transitional B cells in the spleen, before finally differentiating into naïve mature B cells [13]. These cells can further differentiate into three broad populations namely marginal zone B cells, follicular B cells, and plasmablasts (which ultimately become plasma cells), each with specific functional roles in humoral response [13].

While the position of the developmental pathway of Bregs on the classical B cell differentiation process remains unclear, two potential mechanisms have been suggested. The initial hypothesis deemed Bregs as individual lineages of B cells with specific transcription factors (TFs) conferring expression of immunosuppressive genes. The rational coming from other lymphocytes being derived in this manner (such as Tregs and Foxp3) [9]. However, no study has been able to validate the presence of any lineage-specific markers yet which has led to a second theory that B cells, at different developmental stages, acquire regulatory properties in response to environmental stimuli, such as cytokines, inflammation, and cell signalling. This latter hypothesis is supported by studies documenting that immature B cells, mature B cells, and plasmablasts in both humans and mice can differentiate into Bregs in response to stimuli [9]. This is supported by Glass *et al.* using mass-cytometry, and live-cell tracing on *ex vivo* stimulated PBMCs to show that IL-10^+^ human B cells arise from multiple subsets, including CD45RB^+^ CD27^+^ memory, CD24^+^ CD38^+^ transitional, CD19hiCD11c^+^ effector B cells [14]. While immunosuppressive properties of antibody-producing and memory B cells contradict the conventional understanding of their role in inflammation, such regulatory properties of Bregs might be limited to specific subsets of these B cell subpopulations or when these cells are subject to certain stimulatory conditions within the body [15, 16]. This is suggested by the differentiation of regulatory plasmablasts in mice lacking an essential plasma cell transcription factor *BCL6* [15]. Hence, it is important to identify factors inducing differentiation of human Bregs. One transcription factor linked towards immunoregulatory activity in humans is the STAT3 signalling pathway. Shankar *et al*. observed increased STAT3 phosphorylation within their expanded Breg population as well as decreased TIM-1 expression when STAT3 was inhibited [17].

Currently established signals involved in inducing Breg differentiation are predominantly through Toll-like receptor and/or CD40 activation. Toll-like receptors (TLRs) play a crucial role in the development of innate and inflammatory responses by recognizing pathogen-associated molecular patterns (PAMPs) and damage-associated molecular patterns (DAMPs). Stimulation of TLRs in B cells results in differential production of pro-inflammatory and anti-inflammatory cytokines [9, 18]. The significance of TLR signalling in regulating suppressive functions of B cells is evidenced by the development of chronic EAE in mice with B cell-restricted deficiency of TLR signalling molecules MyD88, TLR2, and TLR4 [19]. CD40 signalling is also important for Breg activation; CD40 expressed by B cells binds to CD40 Ligand (CD40L or CD154) expressed by activated T cells, to facilitate B cell differentiation into plasma cells, whereas continuous CD40 signalling limits antibody production [20]. Contradicting conventional beliefs, several studies support the role of CD40 signalling in promoting activation of immunosuppressive Bregs. Mice with CD40-deficient Bregs possess fewer IL10-producing cells, increased T helper type 1 (Th1) and T helper type 1 (Th17) responses, and develop a more severe form of EAE [19]. Indeed, Shankar and colleagues demonstrated that CD154-mediated stimulation of human B cells by co-culture with CD154^+^ CHO cells, could drive differentiation and expansion of IL10^+^ Bregs [17]. Notably, the strength of the CD154 signal determined whether Bregs or activated, non-immunoregulatory B cells were produced. Another important factor in Breg induction is antigen recognition by B cell receptor (BCR), which when impaired inhibits Breg activation and IL-10 production [21], suggesting BCR-dependency in Breg generation.

Differentiation and activation of Bregs have also been shown to be affected by inflammation. This is supported by the increase in Bregs during inflammatory phases of autoimmune conditions, while their absence is associated with more severe episodes of arthritis and EAE in mouse models. For example, one study showed an expansion of Breg populations in arthritic mice in response to proinflammatory cytokines such as IL-1b and IL-6 with their absence leading to worsened symptoms [22]. In addition, stimulating B cells with other inflammatory cytokines, such as IFN-α, IFN-β, IL-21, and GM-CSF, in combination with IL-15, has also shown to facilitate Breg cell differentiation [18, 23]. However, many of these findings were performed in mice, and it is currently unclear whether these observations will apply also to human Bregs.

## Mechanisms of action of Bregs

**Figure 1.**
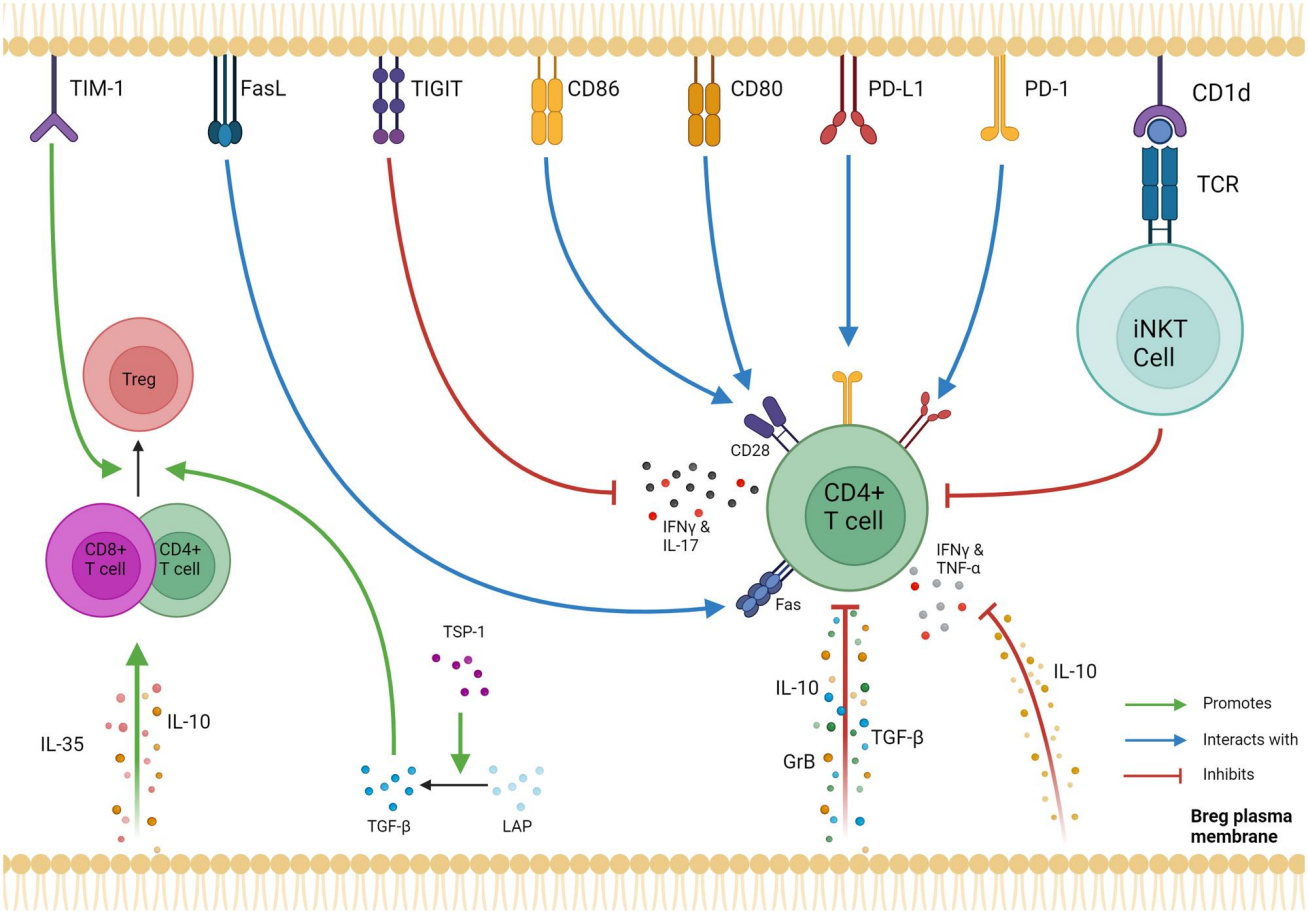
Key mechanisms of Bregs on suppressing other immune cells. (Top) Cell surface receptors expressed by Bregs with known immunosuppressive effects on iNKT and CD4 T cells, and (bottom) secreted factors with known immunosuppressive effects on CD4 and CD8 T cells as well as Tregs. Created in BioRender. Wu, S. (2024) BioRender.com/b10q919.

Bregs have been reported to have a variety of mechanisms of action through both cell surface receptor interactions with target cells as well as through secreted molecules, summarized in Fig. 1 and Table 1.

**Table 1.** Key effector molecules associated with Bregs

Breg gene	Protein	Protein type	Role associated with Bregs
CD1D	CD1d	Membrane	CD1d^+^ marginal zone B cells are involved in the differentiation process of suppressive iNKT cells [24].
CD274	PD-L1/CD274	Membrane	PD-L1^+^ Bregs can bind to PD-1, inhibiting effector T cell activity [25].
CD80	CD80/B7-2	Membrane	mAb-blockade of CD80 and CD86 in co-cultures of CD24^high^CD38^high^ Bregs with CD4^+^ T cells reduced Breg suppressive potency [8].
CD86	CD86/B7-1	Membrane	mAb-blockade of CD80 and CD86 in co-cultures of CD24^high^CD38^high^ Bregs with CD4^+^ T cells reduced Breg suppressive potency [8].
CR1	CR1/CD35	Membrane	Mucosal thromobospondin-1 producing CD35^+^ B cells promote FOXP3^+^ T cell expansion [26].
ENTPD1	NTPDase1/CD39	Membrane	CD39 expression was increased in CpG and IL-2 stimulated B cells within untreated rheumatoid arthritis patients and healthy controls. This also led to suppressed CD4^+^ and CD8^+^ T cell proliferation [27]. CD35 is an inhibitor of BCR-mediated human B cell activation and differentially regulates TLR7, and TLR9 induced responses [28].
GZMB	Granzyme B (GrB)	Secreted/intracellular	GrB produced from Bregs can cleave the ζ-chain from T cell receptor (TCR) inhibiting T cell activation and growth [29] as well as significantly reduce IFNγ and IL-17 expression in T cells [30].
HAVCR1	T-cell immunoglobulin and mucin domain 1 (TIM-1/HAVCR-1)	Membrane	There was significant co-expression of IL-10 and TIM-1 in transitional B cells after CpG and anti-BCR activation. However, the TIM-1^+^ B cells displayed stronger suppressive capabilities than transitional B cells [31]. Agonistic TIM-1 mAbs augment T cell-mediated immune responses, but antagonistic TIM-1 mAbs inhibits immune responses through regulatory B cells [32]. Ex vivo-expanded human CD19^+^ TIM-1^+^ regulatory B cells suppress immune responses *in vivo* and are dependent upon the TIM-1/STAT3 axis [17].
IDO1	Indoleamine 2,3-dioxygenase (IDO)	Intracellular	Over a few days, induced Bregs began to express increased IDO levels (through CTLA-4 action) which also led to increase in FOXP3^+^ T cell proliferation [33].
IL10	Interleukin-10 (IL10)	Secreted/intracellular	The most common immunosuppressive cytokine secreted by Bregs, IL-10 supresses pro-inflammatory cytokines TNF-α and INF-γ and induces conversion of T cells into Tregs [34].
IL12A	Interleukin-12 subunit α (IL-12α)	Secreted/intracellular	Forms a heterodimer with IL-27β as the α subunit to create the regulatory cytokine IL-35 [35].
IL27B/EBI3	Interleukin-27 subunit β (IL-27β)	Secreted/intracellular	Forms a heterodimer with IL-12α as the β subunit to create the regulatory cytokine IL-35 [35].
MME	Membrane metalloendopeptidase (MME/CD10)	Membrane	An increase in CD10^+^ B cells was shown within uncomplicated malaria infection [36].
NT5E	Ecto-5-prime-nucleotidase (NT5E/CD73)	Membrane	Although not an inducible marker, IL-10^-^ B1 cells showed greatly decreased expression of CD73 compared to wild type B1 cells [37]. CD73 is an ecto-5'-nucleotidase that generates adenosine that induces GM-CSF/MDSC-mediated suppression of T cells 35001076 [38].
PDCD1	PD-1/CD279	Membrane	CD4^+^ and CD8^+^ T cells were shown to increase proliferation when an anti-PD-L1 mAb was applied to a coculture of PD-1^+^ Bregs with CD4^+^ and CD8^+^ T cells [39].
PLXNB2	Plexin B2 (PlxnB2)	Membrane	Sema 4C binding to PlxnB2 was found to be the cause of increased IL10 production to WT levels in Sema4C KO variants of murine B cells when co-cultured with Sema4C^+^ HEK293 cells [40].
SDC1	Syndecan 1 (SDC1/CD138)	Membrane	CD138^+^ plasma cells showed increased expression of IL10 and IL-27β compared to the less mature variant CD138^int^ B cells suggesting regulatory functionality is gained through this maturation process [41]. CD138 suppresses apoptosis in multiple myeloma by activating IGF1 Receptor [42].
SEMA4C	Semaphorin 4C (Sema4C)	Membrane	Sema4C KO murine CD138^+^ B cells show expression of pro-inflammatory IL-4 cytokine as opposed to Sema4C^+^ CD138^+^ B cells which expressed greater levels of IL-10 [40] Sema4C shows significantly greater expression in human tonsillar b cells when stimulated by agonistic anti-CD40 and IL4 [43].
TGFB1	Transforming growth factor β (TGF- β)	Secreted	In melanoma patients, greater numbers of TGF-β^+^ B cells compared to healthy controls. Furthermore, these cells were responsible for increased mediation of T cells to undergo differentiation into FOXP3^+^ T cells [44]..
THBS1	Thrombospondin-1 (TSP-1)	Secreted	THBS1 can bind to Latency Associated peptide (LAP), disrupting domain interactions and exposing its binding site thus converting LAP into its biologically active form TGF- β [45] Mesenchymal Stem Cells were found to induce IL-10 production in B cells via TSP-1 induced TGF-β production [46].
TIGIT	TIGIT/VSIG9	Membrane	TIGIT^+^ memory B cells were shown to directly target expression of IFNγ and IL-17 in CD4^+^ T cells as well as suppress dendritic cell maturation resulting in downstream promotion of IL-10^+^ and suppression of IL-21^+^ and IL-4^+^ CD4^+^ T cells [30].

## Phenotypic diversity of Bregs

Despite the lack of consensus on a marker characteristic to Bregs, several subpopulations with distinct surface markers, effector molecules, and functions, have been reported in both human and mice models. Bregs can be also classified into ontogenic subsets, based on the differences in activation modes and resulting cytokine production and cell surface protein production that influence immune cell function within their environment [47]. Thus far in humans, Bregs have been reported to produce three suppressive cytokines namely, IL-10, IL-35 and TGF-β. These three categories are used more widely in literature to classify Bregs. However, multiple other Breg subpopulations, characterized by expression of surface markers or protease production, have been noted in both humans and mice.

### IL-10-producing Bregs (IL-10^+^ Bregs)

IL-10 is an immunosuppressant cytokine that inhibits the production of IFN-γ and TNF-α by antigen-specific T cells, the release of inflammatory mediators from dendritic cells (DCs) and mononuclear cells, and differentiation of Th17 cells [48–50]. In the absence of a specific marker, expression of IL-10 is commonly used to identify Bregs in both humans and mice. An expansion of Il-10^+^ B cell populations have been noted in patients with autoimmune conditions such as arthritis, multiple sclerosis (MS), Systemic lupus erythematosus (SLE) [51, 52], cancers (e.g. breast cancer, gastric cancer (GC)) [10, 53, 54], and infections (e.g. lymphocytic choriomeningitis virus, and vaccinia virus) [55]. However, several groups have demonstrated that whilst a wide spectrum of stimulatory conditions can induce IL-10 expression by B cells, not all IL-10-producing B cells are necessary regulatory [14, 56, 57]. This has made the characterization of definitive cell surface phenotypes of IL-10^+^ Bregs somewhat challenging.

Multiple IL-10^+^ Breg subsets exist, and we will discuss the main ones here. Tedder’s group functionally defined and enumerated a significant proportion of IL-10^+^ Bregs by their competence to express IL-10 following 5 hours of *ex vivo* stimulation with PMA and ionomycin, referring to such Bregs as ‘B10’ cells. Studies have shown that B10 cells and proB10 cells did not express CD38 and were memory in origin (CD24hiCD27^+^ Bregs in human) [58–60]. However, many other IL-10^+^ Breg populations can be stimulated to produce IL-10 under different conditions, for example CD24^hi^CD38^hi^ Bregs [8] CD24^hi^CD27^+^ [61], CD1dhiCD5^+^ IL-10^+^ B cells in the spleen of mice and humans, CD19^+^CD24^hi^CD27^int^ IL-10^+^ plasmablasts [62, 63] in autoimmune conditions, and CD25hiCD71hiCD73loIgG4^+^ IL-10^+^ B cells during allergic reactions. Interestingly regardless of their surface marker combination, these IL-10^+^ B cells regulate immune responses in different conditions by producing IL-10.

Initially it was hypothesized that IL-10^+^ Bregs differentiate from a pro-IL-10^+^ Breg population in response to BCR stimulation by antigen/other cells [62]. However, the presence of B10 cells at different stages of maturation indicates a multi-lineage nature of Bregs, or that IL-10 production occurs in response to microenvironmental stimuli at different stages of B cell development, evidenced by the phenotypic diversity in surface marker expression of B10 cells [62, 64]. For instance, human IL-10^+^ Bregs can express memory or plasma B cell markers CD27, CD38, and CD148 [56, 63], or alternatively, they can be enriched in the naïve and transitional CD19^+^CD24^hi^CD38^hi^ B cell subset found in peripheral blood [62].

An increase in IL-10^+^ Bregs have been documented in the peripheral blood and tumours of patients with malignancy, including breast, gastric, and oesophageal cancers [10, 54, 65]. B10 cells have been shown to promote tumour growth, as tumour infiltrating CD27^+^ CD10^-^ Bregs inhibited inflammatory cytokine production by effector T cells in GC [53]. Depleting IL-10^+^ B cells can diminish pro-tumour immune responses by enhancing cytolytic abilities of splenic cytotoxic T lymphocyte, and B cells [66]. Elevated levels of IL-10^+^ Bregs are also found in chronic Hepatitis B virus (HBV) and Human Immunodeficiency Virus (HIV) infection where they suppressed virus-specific CD8^+^ T cells [67, 68]. In contrast, B cell depletion or deficiency in EAE mice exacerbated severity and prolonged duration of inflammatory phases, which was recoverable by adoptive transfer of IL-10^+^ Bregs, and is implicative of a protective role in autoimmune conditions [16, 69]. A decrease in IL-10^+^ Breg frequency and/or function has also been seen in patients with Rheumatoid Arthritis (RA), Systemic lupus erythematosus (SLE), and asthma [51, 70, 71]. Together, Bregs have been shown to modulate immune responses to cancer, infections, autoimmune diseases via production of IL-10. These different IL-10^+^ Breg populations are represented across the spectrum of immunological tolerance in transplantation, autoimmunity, inflammation, cancer and infection. We are yet to identify a common transcription factor or signature of all IL-10^+^ Bregs. This may be because there are multiple lineages, or indeed because this immunoregulatory function is multi-faceted and dependent on environmental factors.

### IL-35-producing Bregs (IL-35^+^ Bregs)

IL-35 belongs to the IL-12 cytokine family and consists of a β-chain subunit called EBI3 encoded by the *IL27B* gene, and an α subunit called IL-12p35, encoded by *IL12A* gene [72]. IL-35 can regulate the activity of immune cells through the expansion of Tregs, Bregs and suppressing effector T cells, Th1 cells, and Th17 cells, as well as macrophages [41, 73]. The identification of IL-35-producing Tregs that suppressed colitis in mice prompted researchers to explore the existence of IL-35-producing Bregs [43]. Immunoregulatory properties of IL-35^+^ Bregs were noted when treating experimental autoimmune uveitis (EAU) mice with IL-35 resulting in the expansion of IL-10 and IL35-producing Bregs, and suppressed uveitis [35]. This was complemented by Dambuza *et al*.’s findings showing that the transfer of IL-35^+^ Bregs induced an increase in Tregs and IL-10^+^ Bregs, and inhibited proliferation of Th17 and Th1 cells, thereby suppressing EAU [74]. Subsequent studies in humans have shown that the frequency of IL-35^+^ B cells is significantly decreased in SLE, which correlates with reduced plasma IL-35 levels. Further, an abundance of IL-35^+^ Bregs is negatively associated with SLE severity in these patients [75]. A reduction in IL-35^+^ B cell frequency and activity is also seen in ulcerative colitis (UC) [76]. IL-35^+^ Bregs can limit inflammatory T-cell responses in patients in an IL-10-dependent manner, whilst inhibiting anti-microbial immunity through the production of IL-35 [41, 72, 76] and have been shown to play a role in promoting pancreatic cancer [77, 78]. Although several studies have established the immunoregulatory properties of IL-35^+^ Bregs, the mechanisms underlying the suppressive activity of IL-35 remains less understood.

### TGF-β^+^ producing Bregs (TGF-β^+^ Bregs)

Transforming growth factor‐β (TGF‐β) regulates immune responses in different diseases via mediating T cell differentiation, proliferation, and function. Several studies have established the inhibitory roles of TGF -β ^+^ Bregs in humans and mice [79]. These Bregs can be stimulated to secrete TGF-β via multiple pathways, such as the classical signalling involving the TLR, BCR, and CD40 receptors, or growth factor-induced signalling (e.g. phosphatidylinositol-glycan biosynthesis class F protein (PIGF) induced differentiation of TGF-β secreting Bregs in glioma), subjective to the local environment of cells [18]. In mouse models of EAE, a B cell-specific deletion of TGF‐β1, resulted in an earlier onset, greater disease burden, and inflammatory cytokine production, illustrating a role of TGF‐β^+^ Bregs in autoimmune diseases [80]. TGF‐β secreting Bregs have also been shown to modulate immune tolerance in allergic diseases in humans [81]. mAb blocking experiments demonstrated that CpG-stimulated B cells which were initially isolated from human peripheral blood, were able to suppress CD4^+^ T cell proliferation in a TGF‐β-dependent mechanism, whilst promoting the differentiation of Tregs independent of IL-10 [33]. Similarly, TGF-β1^+^ Bregs were found to suppress immune responses in GC patients by mediating the conversion of effector T cells into CD4^+^ Foxp3^+^ Tregs [34].

### CD1d^+^ Bregs

B cells present glycolipids to natural killer T (NKT) cells via CD1d, a non-polymorphic molecule, which subsequently secrete IFN-γ and IL-4 to induce proliferation, maturation and antibody production by B cells [18]. Following presentation of lipid by CD1d to the invariant T cell receptor (iTCR), invariant NKT (iNKT) cells proliferate, produce cytokines and exert cytotoxic effects to regulate innate and adaptive immune responses [82]. NKT cells are reported to enhance antitumor immunity, protection against infections and regulation of autoimmunity [83]. Within human B cells, CD1d is predominantly expressed in Marginal Zone (MZ) -like B cells, IL-10^+^ Bregs and, to a lesser extent in naïve and memory B cells. Early studies noted upregulated CD1d expression in splenic IL-10^+^ B cell subsets in murine models, which correlated with colitis. Indeed, the majority of murine B10 Bregs express high levels of CD1d [84]. Further, CD1d^+^ Bregs suppressed intestinal inflammation in mice with colitis, in an IL-10-dependent manner [5]. Depletion of CD1d^+^ Bregs exacerbated arthritis in mouse models and reduced responses to treatment with α-galactosylceramide, the iNKT cell glycolipid agonist [24]. Oleinka *et al.* described a role for CD1d^+^ Transitional 2-Marginal Zone Precursor (T2-MZP) Bregs in the differentiation of suppressive iNKT cells that inhibited pro-inflammatory Th1 and Th17 responses in the context of experimental arthritis, partially via the production of interferon (IFN)-γ. These mechanisms were IL-10-independent. Conversely, abnormalities in human and mice NKT cells have also been attributed to dysfunctions in CD1d^+^ Bregs. Reductions in CD1d levels may potentially mediate the development of SLE through this mechanism [18, 85].

### PD-1/PD-L1^+^ Bregs

PD-1 is a transmembrane receptor that engages with ligands PD-L1, and PD-L2 to down-modulate immune cell responses. The significance of these interactions is highlighted by the frequent expression of these genes in DCs, NKT cells, B cells and T cells as well as the success of therapeutic approaches such as PD-1 inhibitors in treating cancers, and consequent autoimmune side effects in some patients [86]. PD-L1^+^ Bregs were found to inhibit proliferation of CD8^+^ T cells, CD4^+^ CD25^-^ T cells and CD49b^+^ NK cells in a murine model, via reduced IFN-γ and TNF-α secretion, which ultimately promoted tumour growth in murine breast cancer models [87]. Further, expression of PD-1 and PD-L1 were found to confer immunosuppressive properties to different B cell subsets, in differentiated thyroid cancers (DTC), HCC, and other solid tumours [25, 39, 88]. Expansion of both PD-1^+^ Bregs and PD-L1^+^ Bregs has been found to coincide with advanced disease stage and progression in malignancies [25, 39]. Conversely, decreased expression of PD-L1 in Bregs have been associated with increased severity of allergic rhinitis and food allergies [89].

### Granzyme B (GrB^+^) secreting Bregs

Bregs can modulate immune responses via secretion of cytokines and cytotoxic proteases such as GrB. IL-21 stimulation induced GrB^+^ human Breg, which expressed the phenotype CD19^+^ CD38^+^CD1d^+^ IgM^+^CD147^+^ together with upregulation of IL-10 expression. Analysis of multiple tumour types revealed tumour infiltrating GrB^+^ Bregs adjacent to IL-21-secreting Treg, suggesting a mechanism within the tumour microenvironment [29]. Further, immunoregulatory properties of GrB^+^ Bregs have been inferred due to altered frequencies in association with infections, vaccination, and transplantation. For instance, the peripheral blood of untreated HIV^+^ patients demonstrated CD4^+^ T cells with enhanced IL-21 expression and high *in vivo* frequencies of suppressive GrB^+^ Bregs. When isolated from these patients, co-culture of Il-21^+^ CD40L^-^ Th1 cells with B cells induced GrB^+^ Bregs [90]. Interestingly, the supplementation of CD40L multimers to these co-cultures redirected B cell differentiation toward plasma cells, indicating that CD40L determined the fate of IL-21–dependent B cell differentiation to either regulatory or effector functions.

Similarly, increased frequencies of GrB^+^ Bregs were identified in the peripheral blood of kidney transplant recipients who were operationally tolerant—that is, recipients who did not require pharmacological immunosuppression to maintain stable graft function. GrB expression by Bregs was again dependent on IL-21 stimulation by Th1 cells [91]. In contrast, decreased frequency and dysfunction of GrB^+^ Bregs negatively correlated with disease severity in patients with Rheumatoid Arthritis (RA) [92]. Bregs can secrete GrB to regulate immune responses across a spectrum of immune-mediated disorders.

### Tim-1^+^ Bregs

Tim-1 is an type I membrane protein with an IgV domain followed by a heavily glycosylated mucin domain, a transmembrane domain and an intracellular cytoplasmic tail with one tyrosine phosphorylation motif [93]. TIM-1 is a T-cell co-stimulatory molecule which regulates CD4^+^ T effector cell differentiation and responses in autoimmune and alloimmune settings [32, 94]. Whilst TIM-1 can function as a co-stimulatory molecule for T cell activation [95], it is also important in modulating the function of regulatory T cells (Tregs) [96, 97]. More recently, a role in B cell behaviour has been identified: ligation of TIM-1 induces IL-10 production by TIM-1^+^ B cells and, in so doing, may promote immune tolerance [32].

Using mice which harboured a loss of function TIM-1 mutant, Kuchroo’s group demonstrated that defects in TIM-1 signalling within B cells resulted in reduced IL-10 production by Bregs and severe multi-organ tissue inflammation. Tim-1-deficient B cells promoted Th1 and Th17 responses and inhibited the generation of Tregs, resulting in an increased severity of experimental autoimmune encephalomyelitis (EAE) [98]. Whilst the mechanisms underpinning human TIM-1^+^ Bregs are less well characterized, studies have shown that TIM-1^+^ B cells are more regulatory than TIM-1^-^ B cells in an IL-10-dependent manner [99], and TIM-1 can serve as a marker for a human IL-10^+^ Breg subpopulation which is partially overlapping with transitional B cells [31]. TIM-1^+^ Bregs have been shown to be reduced in frequency and altered in function in autoimmune diseases including systemic sclerosis [31] and T1D [100], but are enriched in cutaneous squamous cell carcinoma (SCC) [17]. Recent work has shown that *ex vivo*-expanded human CD19^+^ TIM-1^+^ Bregs suppress immune responses *in vivo,* they showed that by blocking TIM-1 activity they could significantly reduce the suppressive capability of the Bregs much more than when blocking IL-10 [17]. Although most mechanistic studies are performed in mice, studies have shown that targeting TIM-1 enhances type I interferon (IFN-I) responses, promotes B cells antigen presentation and activation, and enhances T cell anti-tumour responses, thus inhibiting tumour growth [101]. There is now a growing excitement around using TIM-1 as a potential target in immunotherapy [102].

## Functional roles of Bregs in human health and disease

**Figure 2.**
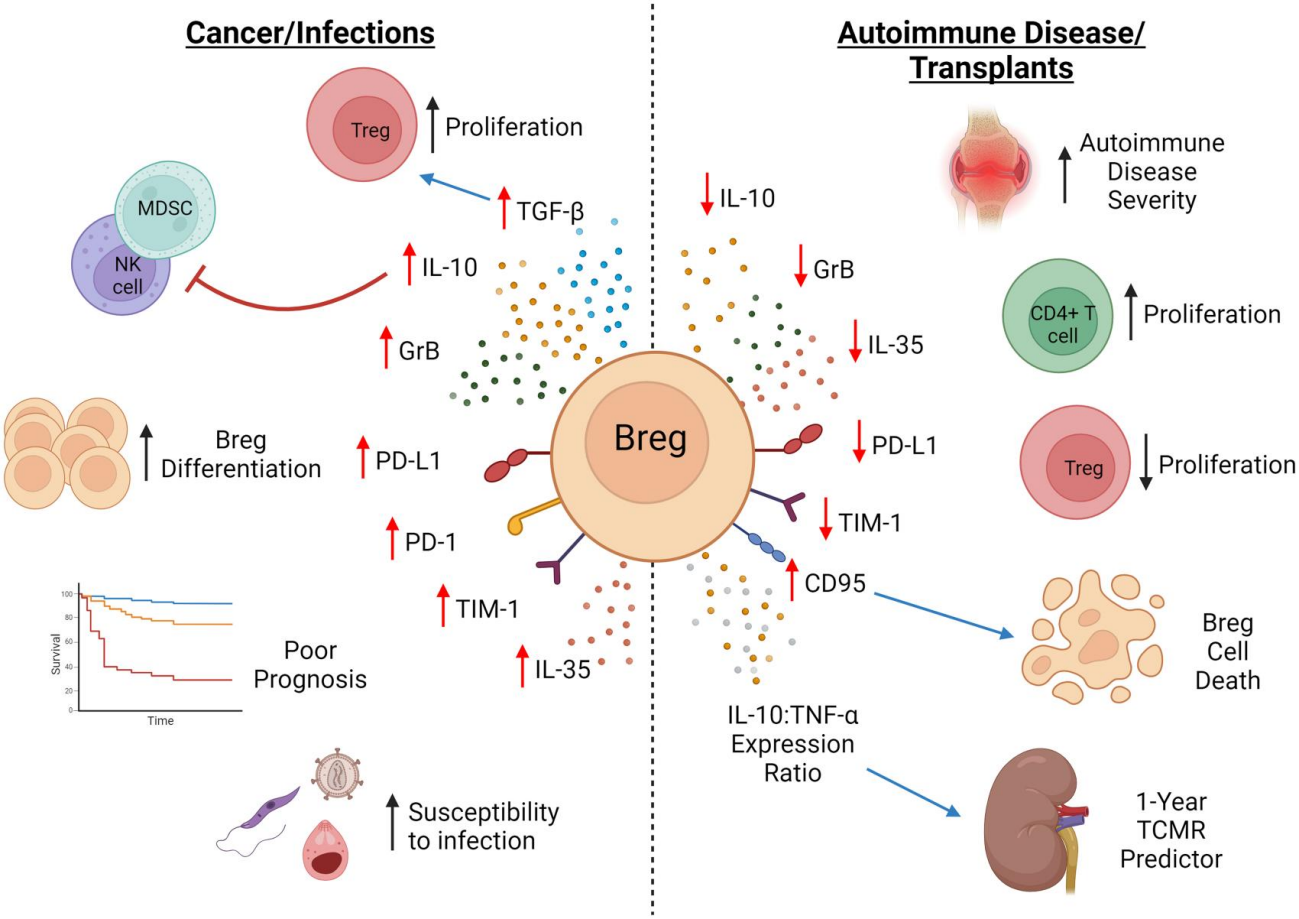
Mechanisms of Breg responses in cancer, infection, autoimmune conditions and transplantation. In cancer and infections, an expansion of IL-10^+^ Bregs and TGF-β secreting Bregs mediate the conversion of effector T cells into Tregs and inhibit the activation of other effector T cell populations. In contrast, a reduction or dysfunction of Bregs have been shown in autoimmune conditions and transplantation responses, where IL-10^+^ Bregs inhibit the secretion of proinflammatory cytokines by Th1, Th17, and monocytes. Further, Bregs inhibit expansion of T cells and promote differentiation of Tregs. IL-10^+^ Bregs can hinder the anti-tumour responses of CD4^+^ and CD8^+^ T cells by curbing their generation of inflammatory cytokines. TGF-β+ Bregs can also induce CD4^+^ T cells to become Tregs. TGF-β- and GrB-secreting Bregs inhibit proliferation of CD4^+^ T and CD8^+^ T cells. Bregs express surface molecules including PD-1 which bind to T cells and limit their anti-tumoural responses. Created in BioRender. Wu, S. (2024) BioRender.com/s75v103.

As previously mentioned, immunoregulatory mechanisms of Bregs have been explored and elucidated in several cancers, infections, and autoimmune conditions. While the expansion of immunosuppressive Bregs promotes immune escape of tumour cells and increases severity of cancers, and infections; they are essential in limiting autoimmune conditions and ensuring transplant tolerance. Table 2 provides an overview of literature in human Bregs in autoimmune diseases, infections, and cancer, summarized in Fig. 2.

**Table 2.** Phenotypic diversity of Bregs in autoimmune conditions, cancer and infections

Disease	Surface marker	Effector molecule	Findings	Reference
Pancreatic Cancer (PC)	CD19^+^ IL-10^+^ Bregs	IL-10, IL-18	Elevated frequencies of CD19^+^ IL-10^+^ Bregs in PC patients versus. healthy controls, which correlated with tumour-node-metastasis (TNM) stage and poor survival. Increased plasma levels of pro-oncogenic IL-18 and increased IL-18R expression by IL10^+^ Bregs of PC patients.	[110]
Ulcerative colitis (UC)	CD1d^+^ IL-10^+^	IL-10	Decreased frequency in CD19^+^ CD1d+IL-10^+^ B cells in UC patients, and impaired IL-10 production *in vitro*.	[111]
Human schistosomiasis	CD1dhi CD24hi CD27+	IL-10, TGF-β	Increased frequency of IL-10^+^ Bregs with enhanced expression of IL-10, latency-associated peptide (LAP) of TGF-β complex in infected patients. Bregs facilitated the conversion of effector T cells to CD25hiFoxP3+Tregs, and increased IL-10 production.	[112]
Hepatitis C virus (HCV) related HCC	CD24+/hi CD38+/hi	IL-10, IL-35	Higher frequency of Bregs in HCC patients than controls, and positively correlation with disease stage, serum IL-10 and IL-35 levels.	[113]
HCC	CD24^+^ CD38+	IL-10, TGF-β1	Increased frequency of CD24^+^ CD38^+^ Bregs in tumour margin and blood coincide with cancer progression. Bregs induced proliferation and invasion of HCC cells via CD40-CD154 signalling.	[114]
Acute Myeloid Leukaemia (AML)		–	Breg frequency was higher in PBMC and BMMC of AML patients, and coincided with advanced disease stage, poorer prognosis and progression.	[115]
Invasive Breast Cancer		IL-10, PD-1	IL-10^+^ CD24^+^ CD38^+^ Bregs was elevated in peripheral blood and tumours of IBC patients compared to healthy controls. Peripheral CD19^+^ B cells from IBC patients but not healthy controls could induce CD4^+^ CD25+Foxp3+Tregs during co-culture in a PD-1-dependant manner.	[116]
Lupus Nephritis (LN)	CD24hi CD38hi	IL-10	Decreased frequency of IL-10^+^ CD19^+^ CD24hiCD38hi Bregs in SLE patients, particularly in LN.	[51]
HIV	CD24hiCD27+, CD24hiCD38hi	IL-10	An increase in frequency of IL-10^+^ Bregs correlated with Tregs in untreated HIV patients which reduced upon treatment.	[68]
Non-Small Cell lung cancer (NSCLC)	CD24hiCD38hi	IL-10	Co-culture of Bregs with tumour-infiltrating follicular cytotoxic CD8^+^ T (Tfc) cells increased Tfc expression of IL-10 and reduced Tfc expression of pro-inflammatory IFN‐γ, TNF, and IL-2 in NSCLC patients.	[117]
Gastric Cancer (GC)		IL-10	Increased % of peripheral IL-10^+^ CD19^+^ CD24hiCD38hi Bregs in GC patients compared to healthy cohort, which correlated with tumour stage. TIL-Breg frequency was enriched compared to peripheral blood in GC. Bregs inhibited secretion of IFN-γ and TNF- α by CD4^+^ T cells, and induced CD4^+^ FoxP3+ Treg differentiation	[34]
HBV		IL-10	In chronic HBV infection, IL-10^+^ Bregs frequency is increased in patients, and are temporally associated with peaks in viral load and hepatic inflammation. In vitro depletion of Bregs led to an increase in virus-specific CD8^+^ T cells.	[67]
HIV		IL-10	HIV^+^ individuals have higher frequency of IL-10^+^ Bregs. Frequency of Bregs is positively associated with viral load and T cell exhaustion. Bregs impair proliferation of CD8+T cells in an IL-10-dependent manner, which can be rescued by Breg depletion.	[118]
Systemic Lupus Erythematosus (SLE)		IL-10	CD19^+^ CD24hiCD38hi Breg-mediated inhibition of T helper (Th1) cell differentiation is modulated by IL-10, CD80, and CD86 signalling.	[8]
Type 1 Diabetes (T1D)		IL-10	IL-10 production by Bregs is significantly reduced in T1D patients.	[119]
Rheumatoid Arthritis (RA)		Il-10	RA patients have reduced number of CD19^+^ CD24hiCD38hi B cells compared to a healthy cohort. Bregs from RA patients failed to induce conversion of CD4^+^ T cells into Treg or to curb Th17 development.	[120]
Rheumatoid Arthritis (RA)	CD24hiCD38hi, CD24hi CD27^+^, CD5^+^, B10	IL-10	Decreased frequency of IL-10^+^ B cells in RA patients compared to a healthy cohort, with inverse correlation to disease severity. B10 cells from RA patients fail to induce differentiation of Tregs.	[121]
Melanoma	CD27^−^ IgM^+^ IgD^+^	PD-L1	Frequency of PD-L1^+^ Bregs was greater in metastatic melanoma patients, and positively correlated with disease stage. PD-L1+ naïve B cells inhibited effector T cell functions in tumours.	[25]
Gastric Cancer (GC)	CD27^+^ CD10^−^	IL-10, CD80/86	IL-10^+^ CD27^+^ CD10 − Bregs were enriched in TIL-B cells, which reduced inflammatory cytokine production by CD4^+^ T cells and CD8^+^ T cells in an IL-10 and CD80/CD86-dependent manner.	[53]
COVID-19	CD27^+^ CD24hi	IL-10	IL-10^+^ Breg population frequency is diminished in patients with critical and severe COVID-19, and corresponds to a hyperinflammatory response marked by increase in clinical inflammatory parameters including neutrophil/lymphocyte ratio, D-dimer presence etc in patients.	[109]
Differentiated Thyroid Cancer (DTC)	CD27^+^ IL-10+	IL-10	Enrichment of TIL-Bregs correlated with higher frequency of Foxp3+Tregs within tumours of DTC patients. Peripheral CD27^+^ Bregs from DTC patients produced more IL-10 than those from healthy cohort, and inhibited IFN-γ expression by CD4^+^ T cells, perforin and GrB expression by CD8^+^ T cells.	[122]
Breast, ovarian, cervical, colorectal, prostate carcinomas	CD38^+^ CD1dhi IgM^+^ CD147+	GrB	IL-21 induced GrBhi Breg is enriched in tumour tissue and promotes GrB-dependent degradation of TCR, inhibiting T cell proliferation.	[29]
Head and Neck cancer	CD39^+^ CD73+	Adenosine (ADO)	Frequency of ADO producing Bregs was significantly decreased in tumour microenvironment compared to PBMC, but created an immunosuppressive environment.	[123]
Ulcerative colitis (UC)	CD5^+^ CD24hiCD38hi	IL-10	Lower number of CD5^+^ and CD24hiCD38hi Bregs and increased in CD95^+^ exhausted B cells in UC patients.	[124]
Allergic Asthma	CD5^+^ CD1d+	IL-10	Decreased frequency of CD5+and CD1d^+^ CD5+transitional B cells in patients with asthma. Oral corticosteroids inhibited IL-10 production by these cells in patients.	[125]
Oesophageal squamous cell carcinoma (ESCC)		IL-10	Increased frequency of IL-10^+^ Bregs in ESCC patients versus. healthy cohort. Exosomes isolated from peripheral blood of ESCC patients could induce differentiation of IL-10^+^ B cells and PD1^+^ Bregs.	[126]
Cervical Cancer (CC)		IL-10	Frequency of CD19^+^ CD5^+^ CD1d^+^ Bregs and serum IL-10 level was higher in CC patients. Bregs were strongly associated with disease progression and metastasis, but negatively correlated with CD8^+^ T cell frequency.	[127]
Systemic Lupus Erythematosus (SLE)	CD5^+^ CD1dhigh	IL-10	Increased frequency of CD19^+^ IL-10^+^ CD5^+^ CD1dhigh Bregs in SLE, particularly during inflammatory phases. Frequency decreased during disease remission.	[104]
HCC	CD5hiCD24−/+CD27hi/+CD38dim	PD-1	Higher frequency of PD-1hi Bregs exhibiting CD5hiCD24−/+CD27hi/+CD38dim phenotype in HCC tissue. PD-1hi Bregs positively associated with disease progression and recurrence, and plasma IL-10 levels.	[88]
Hepatocellular Carcinoma (HCC)	CD5hiCD24 − CD27−/+CD38+/hi TIM-1+	TIM-1/HAVCR1	Proportion of TIM-1^+^ Bregs was elevated in tumours compared to peri-tumoural regions and blood of cancer patients, and positively correlated with disease progression, early recurrence, and invasion. TIM-1^+^ Breg frequency was negatively correlated to overall survival and limited proliferation and function of CD8^+^ effector T cells. TIM-1^+^ Bregs facilitated immune escape in HCC via novel exosomal HMGB1-TLR2/4-MAPK pathways.	[128]
Rheumatoid Arthritis (RA)	PD-L1+, CD24hiCD38 − PD-L1+, CD24hiCD38hiPD-L1+	PD-L1	Decreased frequency of PD-L1^+^ Bregs in RA patients. % PD-L1^+^ Bregs increases in response to treatment. PD-L1^+^ Bregs suppress CD8^+^ T cells proliferation and cytokine production in vitro.	[129]
HIV	TIM-1+	IL-10	IL-10^+^ Bregs increase in early HIV-1 infection in human and mice. Bregs suppress anti-HIV-specific T cell responses in an IL-10-dependent manner.	[130]

### Bregs in auto-immune diseases

In the context of immune diseases, human Bregs were first discovered in SLE where CD19^(+)^CD24^(hi)^CD38^(hi)^ B cells exhibit regulatory capacity in healthy individuals but are functionally impaired in SLE patients [8]. Later studies established the role of IL-10-producing Bregs in immune suppression and maintenance of tolerance, typically impaired in autoimmune diseases. The role of Bregs in autoimmune conditions such as SLE or RA is complicated by the variety of phenotypes, frequencies, and functional roles of the Breg populations described [103]. However, extensive validation within mouse models point to Bregs as an exciting therapeutic option for these autoimmune disorders.

An abundance of CD19^+^ CD5^+^CD1d^hi^ Bregs, and CD19^+^CD24^hi^CD38^hi^ Bregs were observed in active SLE patients in two separate studies [8, 104]. Contradictions on unchanged or reduced numbers of CD19^+^CD24^hi^CD38^hi^ cells in SLE patients have also been noted. Blair *et al.* reported a significantly higher frequency of CD19^+^CD24^hi^CD38^hi^ Bregs in PBMCs of patients, but a comparable absolute proportion. They proposed that a functional defect in CD19^+^CD24^hi^CD38^hi^ B cells was involved in SLE pathogenesis [8]. This is supported by a refractory response to CD40 stimulation and reduced IL-10 production by CD19^+^CD24^hi^CD38^hi^ B cells isolated from SLE patients [8]. The discrepancies in findings highlighted above limit our understanding of the mechanistic role of Bregs in SLE pathogenesis, potentially resulting from differences in cell stimulation approaches, IL-10 production levels of the cell population, and stage of diseases in samples assessed. It does not imply absence of the mechanistic role of Bregs in SLE [103]. As discussed in previous sections, Bregs can also modulate immune responses by IL-10-independent mechanisms. This is exemplified by Menon *et al.* [105] demonstrating the regulatory feedback mechanism between plasmacytoid DCs and Bregs via the release of IFN-α and CD40 engagement, which is altered in SLE leading to impaired Breg differentiation. Functional and/or numerical deficiencies in Bregs have also been found in patients with other autoimmune diseases, including Type 1 Diabetes (T1D), Lupus nephritis (LN), ulcerative colitis (UC), detailed in Table 1.

### Bregs in infections

Bregs not only limit immune responses in autoimmune and chronic inflammatory diseases but can also dampen immune responses to infection, consequently increasing disease severity. Phenotypically and functionally distinct Bregs have thus been characterized in bacterial, viral, and parasitic infections [106]. The frequency of CD19^+^CD24^hi^CD38^hi^ Bregs significantly increased in HIV-infected individuals and correlated to active disease. Further, exposure to HIV-1 facilitates the differentiation of B cells into Bregs with an immunosuppressive repertoire of cytokines including high levels of IL-10, TGF-β1, EBI3 or IL-12 (p35) [107]. These cells were also capable of inhibiting effector functions of CD4^+^ T cells and CD8^+^ T cells. Similar expansions of IL-10-producing CD19^+^CD24^hi^CD38^hi^ regulatory B cells in chronic HBV infections, and IL-10^+^ neonatal Bregs in Respiratory Syncytial virus (RSV), demonstrate that IL-10 production by Bregs limits adaptive responses to viral infections [68, 108]. Conversely, a reduction in the frequency of IL-10^+^ Bregs has been associated with hyperinflammatory responses in critical and severe COVID-19 patients, supported by an increase in clinical inflammatory parameters, including neutrophil/lymphocyte ratio and the D-dimer presence [109]. An expansion of IL10^+^ Bregs were also associated with greater susceptibility to cerebral malaria, and Leishmania [36]. Multiple studies have demonstrated the role of Bregs in murine models of infectious diseases, the implications of which can be extended to gain insights on the mechanistic role of Bregs in human infections.

### Bregs in cancer

Phenotypically varied Bregs have been shown to suppress immunity and promote tumour growth in several cancers. The expansion of IL-10^+^, TIM-1^+^, PD-1^+^ and GrB^+^ Bregs has been correlated with progressive disease stage and poorer prognosis in patients, highlighting the intricate functional roles of these cells in cancer pathogenesis, prognosis and outcomes.

An enrichment of IL10^+^ CD19^+^CD24^hi^CD38^hi^ Bregs promoting the conversion of effector T cells into Foxp3^+^ Tregs *in vitro* was identified in breast and gastric cancer patients [34, 116]. IL-10^+^ Bregs dampened the production of pro-inflammatory cytokines IFN- γ, TNF, and IL-17 by CD4^+^ T cells and CD8^+^ T cells [53, 131]. As described in earlier sections, B cells can also impair immune responses in cancer by secreting cytotoxic proteases such as GrB. Mechanistic exploration demonstrated that IL-21-mediated induction of GrB was dependent on both BCR and TLR signaling pathways, illustrating tight regulation of GrB^+^ Breg induction. Elevated IL-21-induced GrB^+^ Bregs have been found in the tumour microenvironment (TME) of breast, ovarian, cervical, colorectal, and prostate carcinomas [29]. GrB^+^ Bregs limited T-cell proliferation by a GrB-dependent degradation of the T-cell receptor ζ-chain, offering novel therapeutic strategies in cancer [29].

The PD-1/PD-L1 Breg axis also offers therapeutic possibilities in cancer treatments. An increased frequency of tumour infiltrating PD-1^+^ Bregs was identified in the context of Hepatocellular carcinoma (HCC), which correlated with disease progression, poor survival, and recurrence in patients [88]. Stimulation of tumour-derived PD-1^+^ Bregs with PD-1 Ligand (PD-L1) and subsequent co-culture with tumour-derived effector memory T cells, resulted in impaired proliferation and production of granzymes and perforin by CD8^+^ T cells in an IL-10-dependent mechanism, thereby contributing to tumour growth. Similarly the frequency of circulating and tumour-derived PD-L1^+^ Bregs correlated with disease stage in melanoma patients which were rare in healthy controls [25]. Naive circulating B cells with moderate PD-L1 expression isolated from patients with stage III and stage IV melanoma, could significantly suppress pro-inflammatory T cell responses in a PD-L1-dependent mechanism [25, 116]. Alongside inhibiting effector functions of T cells within the tumour microenvironment, Bregs can also induce differentiation of regulatory T cells (Treg) in a TGF-β-dependent manner [79]. IL-10^+^ Bregs have also been found to promote the suppressive effect of MDSCs and abrogate NK cell-mediated lysis of tumour cells in cancers such as Multiple Myeloma [25, 132, 133]. Overall, Bregs exert their capacity to exert immunosuppressive effects in cancer via multiple mechanisms.

### Bregs in transplantation

B cells are thought to mediate acute and chronic rejection in transplant patients by producing *de novo* and donor-specific antibodies, by presenting antigens to alloreactive T cells and co-stimulating them. Conversely, Bregs are able to modulate the alloimmune response by multiple mechanisms including the secretion of immunosuppressive cytokines, to improve outcomes in experimental models [134]. Adoptive transfer studies have demonstrated that an array of Breg subsets can prolong allograft survival and promote transplant tolerance in an antigen-specific manner, often dependent on multiple mechanisms including IL-10, TIM-1, TGFβ, GrB and the induction of Tregs [134–137].

The rarity of human Bregs together with challenges in defining specific human Breg subsets and phenotypic discrepancies across species have made the clinical translation of these findings challenging. Depletion of B cells in kidney transplant recipients (KTRs) by Rituximab therapy accelerated rejection in transplant patients, first implicating regulatory B cell involvement in transplant tolerance [7]. Later studies showed that operationally tolerant KTRs (long-term stable graft function without the need for immunosuppressants) had increased frequencies of circulating Bregs compared to KTRs with stable graft function with immunosuppressants or those undergoing T cell mediated rejection (TCMR) [91]. The Immune Tolerance Network have reported similar findings in two USA and European-wide consortia studies, whilst also identifying B cell-based gene signatures specific to transplant tolerant patients [134, 138]. Cherukuri *et al.* has gone on to demonstrate that within circulating CD24^hi^CD38^hi^ transitional B cells of KTRs, that the ratio of IL-10/TNF-α expression serves as an accurate predictor of 1-year rates of TCMR as well as graft function and graft survival at 5 years post-transplantation [139]. These observations not only suggest markers which could be used in immunosuppression minimization trials in transplantation, but also illustrate that perhaps the balance of Bregs to B effector subsets, rather than simply absolute frequencies, may be an important element in determining clinical outcomes [140]. Recent advances in human Breg expansion protocols have also demonstrated the therapeutic possibilities of adoptive Breg cell therapy in a clinically relevant humanized mouse model of human skin transplantation [17]. Insights from such work may pave the way to translation for adoptive cell therapy in the clinic.

### Opportunities and outstanding challenges in the clinical applications of Bregs

**Figure 3.**
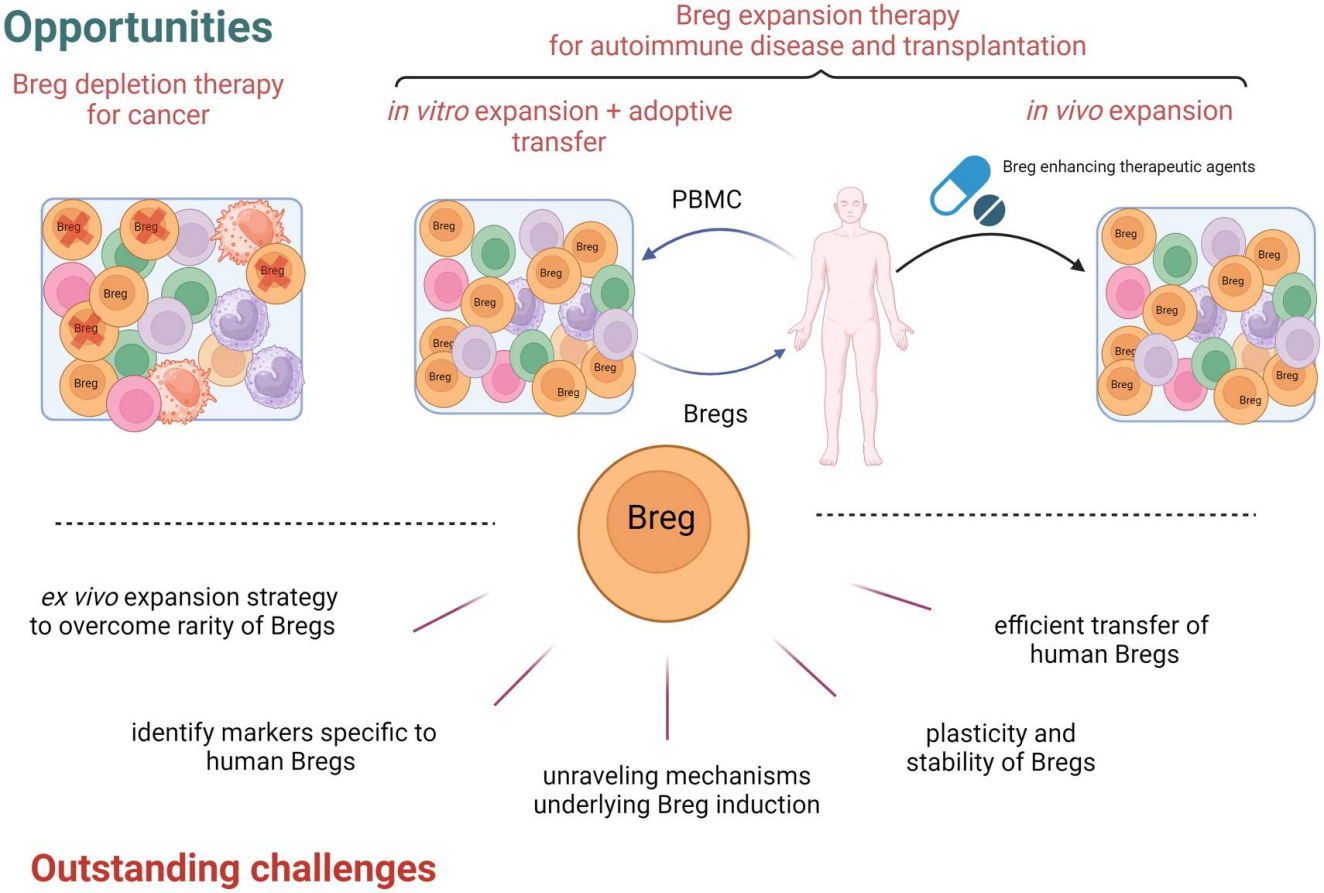
Opportunities and challenges of Bregs. Bregs could be targeted for depletion to augment anti-tumour immune responses. In vitro expansion of patient-derived Bregs and adoptive transfer into patients could suppress autoimmune or alloimmune responses in patients with autoimmune disorders or transplants. Boosting endogenous Breg populations via therapeutic agents could promote Breg expansion and induce tolerance in these patients. Created in BioRender. Wu, S. (2024) BioRender.com/a43i376.

The immunosuppressive properties of Bregs have been shown to play significant roles across autoimmune diseases, cancers, and infections, which highlights their potential in clinical settings. Here, we identify several such opportunities (Fig. 3).

### Breg depletion in cancer

Emerging research suggests that Bregs might be manipulated to enhance cancer immunotherapy approaches by modulating the immune response to tumours. As discussed in earlier sections, increased Breg frequency is associated with diminished anti-tumoral immune responses in cancer patients. These observations offer fresh therapeutic possibilities in the form of novel targets for the depletion or inhibition of Bregs which may reactivate immune responses to cancer. Such strategies are already being explored. The inhibition of Mitogen/extracellular signal regulated kinase (MEK) using Cobimetinib in a pre-clinical *in vivo* colorectal cancer model, resulted in decreased Breg frequency within the draining lymph node, improved T cell infiltration and an enhanced response to anti-PD1 immunotherapy [141]. While Breg depletion by anti-CD20 antibodies has been explored to treat cancers, this approach inevitably has off-target effects when targeting a pan-B cell marker, thus resulting in concurrent depletion of anti-tumour B cell subsets [142]. Further studies investigating the depletion of IL-35-producing Bregs to treat infections and cancers are underway [72]. In addition, there is the potential for the use of Breg phenotypes as a biomarker of treatment response. For example, increase of IL-10^+^ Bregs isolated from melanoma patients pre-treatment, positively correlated with a lack of response to anti-CTLA-4 treatment, indicating the potential to utilize IL-10^+^ Bregs as a biomarker for treatment response [143]. Definitive clarity of Breg phenotypes will be required to develop precision-based targeting for effective, oncological therapies.

### Breg therapy in autoimmune diseases, inflammatory disorders, and transplantation

Debilitating autoimmune conditions such as EAE, chronic colitis, and CIA, can be experimentally ameliorated by adoptive transfer of Breg subsets in animal models, thus indicating a potential therapeutic opportunity [62]. Harnessing the regulatory functions of Bregs could lead to new therapeutic strategies for managing these inflammatory conditions and in the setting of transplantation, and potentially could be harnessed to control excessive inflammation in conditions like sepsis.

### In vitro expansion of Bregs

Inducing Breg populations *in vitro* and subsequently transferring them into patients is an alternative strategy to suppress hyperinflammatory reactions in autoimmune diseases. However, this approach has several challenges including determining signals to induce Breg expansion, developing methods for their effective administration in patients, and ensuring their stability in host systems. Recently, protocols for *ex vivo* expansion and maintenance of human Bregs with *in vivo* immunoregulatory properties in humanized mouse models, have been developed [17]. This opens avenues to overcome the challenges of rare human Breg subsets and will facilitate the development of cell therapies in transplantation and autoimmune conditions. The recent evolution of Chimeric Antigen Receptor (CAR) technology has revolutionized the T cell therapy landscape [144] and may also do the same for Breg cell therapies to ensure potency and to mitigate off-target effects. Future pre-clinical investigations are needed to determine concentration and specificity of stimuli to induce Bregs, the functional stability of a Breg cell therapy product and the appropriate therapeutic dosages required for clinical purposes [145].

### Bregs as a prognostic predictor

Given the regulatory and immunosuppressive functions of Bregs suggesting a role in shaping disease outcomes, Bregs have emerged as potential prognostic predictors in various diseases. As described above, many studies have shown that Breg frequencies and functional properties correlate with disease severity and prognosis across multiple disease types. For example, the ratio of IL-10 to TNFα produced by transitional-1 B cells 3 months after transplantation was shown to be a predictive biomarker for clinical and subclinical renal allograft rejection and subsequent clinical course [140]. Investigating the presence and activity of Bregs as prognostic indicators holds promise in tailoring treatment strategies and predicting disease trajectories, contributing to a more personalized approach to patient care.

Challenges persist in standardizing Breg phenotyping and characterizing their specific roles in different disease contexts, necessitating further research and validation. There is no unique surface marker to distinguish them from other B cell subtypes. Bregs are heterogeneous, and their functions can vary. Understanding this diversity and developing approaches to harness specific Breg subtypes will be fundamental to harnessing their translational potential. The precise mechanisms by which Bregs regulate immune responses are not fully understood, making it difficult to develop targeted therapies. The enduring effects of manipulating Bregs and their long-term stability in the context of the wider immune milieu remain to be seen. Finally, developing standardized protocols for Breg isolation, expansion, and administration will be crucial to facilitate translation.

## Future directions and conclusion

B cells are well-characterized contributors to the adaptive and humoral immune responses. The discovery of B cells with immunosuppressive roles across a spectrum of immune-mediated diseases has unveiled novel insights into B cell behaviour in human health and disease. Studies conducted over the last few decades have significantly expanded our understanding of the immunobiology of Bregs and their role in the pathophysiology of cancers, autoimmune diseases, transplantation and infectious diseases. The therapeutic potential presented by these advances in Breg biology are currently limited by a lack of unique phenotypic signatures to identify Bregs. An incomplete understanding of the origin and differentiation of Bregs from B cells in response to pathogenic conditions further constrains efforts to design effective treatments. Given the growing significance of Bregs in health and diseases, the use of single cell multi-omics data (paired RNA-sequencing, B cell receptor sequencing and proteomics), human-centric functional analyses and standardized experimental conditions may present exciting opportunities by which to advance this field from bench to the bedside.

## Data Availability

There are no new data associated with this article.

## References

[1] Katz SI , Parker D, Turk JL. B-cell suppression of delayed hypersensitivity reactions. Nature 1974;251:550–1. 4547522 10.1038/251550a0

[2] Neta R , Salvin SB. Specific suppression of delayed hypersensitivity: the possible presence of a suppressor B cell in the regulation of delayed hypersensitivity. J Immunol 1974;113:1716–25. 4279260

[3] Kennedy MW , Thomas DB. A regulatory role for the memory B cell as suppressor-inducer of feedback control. J Exp Med 1983;157:547–58. 6185613 10.1084/jem.157.2.547 PMC2186930

[4] Mizoguchi A , Mizoguchi E, Smith RN et al Suppressive role of B cells in chronic colitis of T cell receptor alpha mutant mice. J Exp Med 1997;186:1749–56. 9362534 10.1084/jem.186.10.1749 PMC2199135

[5] Mizoguchi A , Mizoguchi E, Takedatsu H et al Chronic intestinal inflammatory condition generates IL-10-producing regulatory B cell subset characterized by CD1d upregulation. Immunity 2002;16:219–30. 11869683 10.1016/s1074-7613(02)00274-1

[6] Wolf SD , Dittel BN, Hardardottir F, Janeway CA. Jr., Experimental autoimmune encephalomyelitis induction in genetically B cell-deficient mice. J Exp Med 1996;184:2271–8. 8976182 10.1084/jem.184.6.2271 PMC2196394

[7] Clatworthy MR , Watson CJE, Plotnek G et al B-cell-depleting induction therapy and acute cellular rejection. N Engl J Med 2009;360:2683–5. 19535812 10.1056/NEJMc0808481 PMC4143588

[8] Blair PA , Noreña LY, Flores-Borja F et al CD19(+)CD24(hi)CD38(hi) B cells exhibit regulatory capacity in healthy individuals but are functionally impaired in systemic Lupus Erythematosus patients. Immunity 2010;32:129–40. 20079667 10.1016/j.immuni.2009.11.009

[9] Rosser EC , Mauri C. Regulatory B cells: origin, phenotype, and function. Immunity 2015;42:607–12. 25902480 10.1016/j.immuni.2015.04.005

[10] Sarvaria A , Madrigal JA, Saudemont A. B cell regulation in cancer and anti-tumor immunity. Cell Mol Immunol 2017;14:662–74. 28626234 10.1038/cmi.2017.35 PMC5549607

[11] Mohd Jaya FN , Garcia SG, Borras FE et al Paradoxical role of Breg-inducing cytokines in autoimmune diseases. J Transl Autoimmun 2019;2:100011. 32743499 10.1016/j.jtauto.2019.100011 PMC7388338

[12] Fridman WH , Petitprez F, Meylan M et al B cells and cancer: To B or not to B? J Exp Med 2020;218:e20200851. 10.1084/jem.20200851 PMC7754675 33601413

[13] Pieper K , Grimbacher B, Eibel H. B-cell biology and development. J Allergy Clin Immunol 2013;131:959–71. 23465663 10.1016/j.jaci.2013.01.046

[14] Glass MC , Glass DR, Oliveria J-P et al Human IL-10-producing B cells have diverse states that are induced from multiple B cell subsets. Cell Rep 2022;39:110728. 35443184 10.1016/j.celrep.2022.110728 PMC9107325

[15] Matsumoto M , Baba A, Yokota T et al Interleukin-10-producing plasmablasts exert regulatory function in autoimmune inflammation. Immunity 2014;41:1040–51. 25484301 10.1016/j.immuni.2014.10.016

[16] Matsushita T , Horikawa M, Iwata Y, Tedder TF. Regulatory B cells (B10 cells) and regulatory T cells have independent roles in controlling experimental autoimmune encephalomyelitis initiation and late-phase immunopathogenesis. J Immunol 2010;185:2240–52. 20624940 10.4049/jimmunol.1001307 PMC3717968

[17] Shankar S , Stolp J, Juvet SC et al Ex vivo-expanded human CD19(+)TIM-1(+) regulatory B cells suppress immune responses in vivo and are dependent upon the TIM-1/STAT3 axis. Nat Commun 2022;13:3121. 35660734 10.1038/s41467-022-30613-z PMC9166804

[18] Catalán D , Mansilla MA, Ferrier A et al Immunosuppressive mechanisms of regulatory B cells. Front Immunol 2021;12:611795. 33995344 10.3389/fimmu.2021.611795 PMC8118522

[19] Neves P , Lampropoulou V, Calderon-Gomez E et al Signaling via the MyD88 adaptor protein in B cells suppresses protective immunity during Salmonella typhimurium infection. Immunity 2010;33:777–90. 21093317 10.1016/j.immuni.2010.10.016

[20] Miyashita T , McIlraith MJ, Grammer AC et al Bidirectional regulation of human B cell responses by CD40-CD40 ligand interactions. J Immunol 1997;158:4620–33. 9144474

[21] Jansen K , Cevhertas L, Ma S et al Regulatory B cells, A to Z. Allergy 2021;76:2699–715. 33544905 10.1111/all.14763

[22] Rosser EC , Oleinika K, Tonon S et al Regulatory B cells are induced by gut microbiota-driven interleukin-1beta and interleukin-6 production. Nat Med 2014;20:1334–9. 25326801 10.1038/nm.3680

[23] Chekol Abebe E , Asmamaw Dejenie T, Mengie Ayele T et al The role of regulatory B cells in health and diseases: a systemic review. J Inflamm Res 2021;14:75–84. 33469337 10.2147/JIR.S286426 PMC7811483

[24] Oleinika K , Rosser EC, Matei DE et al CD1d-dependent immune suppression mediated by regulatory B cells through modulations of iNKT cells. Nat Commun 2018;9:684. 29449556 10.1038/s41467-018-02911-y PMC5814456

[25] Wu H , Xia L, Jia D et al PD-L1(+) regulatory B cells act as a T cell suppressor in a PD-L1-dependent manner in melanoma patients with bone metastasis. Mol Immunol 2020;119:83–91. 32001420 10.1016/j.molimm.2020.01.008

[26] Zhang H-P , Wu Y, Liu J et al TSP1-producing B cells show immune regulatory property and suppress allergy-related mucosal inflammation. Sci Rep 2013;3:3345. 24736213 10.1038/srep03345 PMC4002291

[27] Zacca ER , Amezcua Vesely MC, Ferrero PV et al B cells from patients with rheumatoid arthritis show conserved CD39-mediated regulatory function and increased CD39 expression after positive response to therapy. J Mol Biol 2021;433:166687. 33098857 10.1016/j.jmb.2020.10.021 PMC9376888

[28] Macsik-Valent B , Nagy K, Fazekas L, Erdei A. Complement receptor type 1 (CR1, CD35), the inhibitor of BCR-mediated human B cell activation, differentially regulates TLR7, and TLR9 induced responses. Front Immunol 2019;10:1493. 31312202 10.3389/fimmu.2019.01493 PMC6614493

[29] Lindner S , Dahlke K, Sontheimer K et al Interleukin 21-induced granzyme B-expressing B cells infiltrate tumors and regulate T cells. Cancer Res 2013;73:2468–79. 23384943 10.1158/0008-5472.CAN-12-3450

[30] Hasan MM , Nair SS, O'Leary JG et al Implication of TIGIT(+) human memory B cells in immune regulation. Nat Commun 2021;12:1534. 33750787 10.1038/s41467-021-21413-y PMC7943800

[31] Aravena O , Ferrier A, Menon M et al TIM-1 defines a human regulatory B cell population that is altered in frequency and function in systemic sclerosis patients. Arthritis Res Ther 2017;19:8. 28103916 10.1186/s13075-016-1213-9 PMC5248463

[32] Ding Q , Yeung M, Camirand G et al Regulatory B cells are identified by expression of TIM-1 and can be induced through TIM-1 ligation to promote tolerance in mice. J Clin Invest 2011;121:3645–56. 21821911 10.1172/JCI46274 PMC3163958

[33] Nouël A , Pochard P, Simon Q et al B-cells induce regulatory T cells through TGF-beta/IDO production in A CTLA-4 dependent manner. J Autoimmun 2015;59:53–60. 25753821 10.1016/j.jaut.2015.02.004

[34] Wang WW , Yuan XL, Chen H et al CD19+CD24hiCD38hiBregs involved in downregulate helper T cells and upregulate regulatory T cells in gastric cancer. Oncotarget 2015;6:33486–99. 26378021 10.18632/oncotarget.5588 PMC4741780

[35] Wang R-X , Yu C-R, Dambuza IM et al Interleukin-35 induces regulatory B cells that suppress autoimmune disease. Nat Med 2014;20:633–41. 24743305 10.1038/nm.3554 PMC4048323

[36] Soares RR , Antinarelli LMR, Abramo C et al What do we know about the role of regulatory B cells (Breg) during the course of infection of two major parasitic diseases, malaria and leishmaniasis? Pathog Glob Health 2017;111:107–15. 28353409 10.1080/20477724.2017.1308902 PMC5445636

[37] Kaku H , Cheng KF, Al-Abed Y, Rothstein TL. A novel mechanism of B cell-mediated immune suppression through CD73 expression and adenosine production. J Immunol 2014;193:5904–13. 25392527 10.4049/jimmunol.1400336 PMC4321875

[38] King RJ , Shukla SK, He C et al CD73 induces GM-CSF/MDSC-mediated suppression of T cells to accelerate pancreatic cancer pathogenesis. Oncogene 2022;41:971–82. 35001076 10.1038/s41388-021-02132-6 PMC8840971

[39] Wang X , Wang G, Wang Z et al PD-1-expressing B cells suppress CD4(+) and CD8(+) T cells via PD-1/PD-L1-dependent pathway. Mol Immunol 2019;109:20–6. 30851633 10.1016/j.molimm.2019.02.009

[40] Xue D , Kaufman GN, Dembele M et al Semaphorin 4C protects against allergic inflammation: requirement of regulatory CD138+ plasma cells. J Immunol 2017;198:71–81. 27881703 10.4049/jimmunol.1600831

[41] Shen P , Roch T, Lampropoulou V et al IL-35-producing B cells are critical regulators of immunity during autoimmune and infectious diseases. Nature 2014;507:366–70. 24572363 10.1038/nature12979 PMC4260166

[42] Beauvais DM , Jung O, Yang Y et al Syndecan-1 (CD138) suppresses apoptosis in multiple myeloma by activating IGF1 receptor: prevention by SynstatinIGF1R inhibits tumor growth. Cancer Res 2016;76:4981–93. 27364558 10.1158/0008-5472.CAN-16-0232 PMC5010496

[43] Xue D , Desjardins M, Kaufman GN et al Semaphorin 4C: a novel component of B-cell polarization in Th2-driven immune responses. Front Immunol 2016;7:558. 28003812 10.3389/fimmu.2016.00558 PMC5141245

[44] Harris RJ , Willsmore Z, Laddach R et al Enriched circulating and tumor-resident TGF-β+ regulatory B cells in patients with melanoma promote FOXP3+Tregs. OncoImmunology 2022;11:2104426. 35909944 10.1080/2162402X.2022.2104426 PMC9336482

[45] Murphy-Ullrich JE , Suto MJ. Thrombospondin-1 regulation of latent TGF-beta activation: A therapeutic target for fibrotic disease. Matrix Biol 2018;68-69:28–43. 29288716 10.1016/j.matbio.2017.12.009 PMC6015530

[46] Liu J , Lai X, Bao Y et al Intraperitoneally delivered mesenchymal stem cells alleviate experimental colitis through THBS1-mediated induction of IL-10-competent regulatory B cells. Front Immunol 2022;13:853894. 35371051 10.3389/fimmu.2022.853894 PMC8971528

[47] Baba Y , Saito Y, Kotetsu Y. Heterogeneous subsets of B-lineage regulatory cells (Breg cells). Int Immunol 2020;32:155–62. 31630184 10.1093/intimm/dxz068

[48] Bouaziz JD , Le Buanec H, Saussine A et al IL-10 producing regulatory B cells in mice and humans: state of the art. Curr Mol Med 2012;12:519–27. 22292445 10.2174/156652412800620057

[49] Madan R , Demircik F, Surianarayanan S et al Nonredundant roles for B cell-derived IL-10 in immune counter-regulation. J Immunol 2009;183:2312–20. 19620304 10.4049/jimmunol.0900185 PMC2772089

[50] Ray A , Basu S, Williams CB et al A novel IL-10-independent regulatory role for B cells in suppressing autoimmunity by maintenance of regulatory T cells via GITR ligand. J Immunol 2012;188:3188–98. 22368274 10.4049/jimmunol.1103354 PMC3311743

[51] Heinemann K , Wilde B, Hoerning A et al Decreased IL-10(+) regulatory B cells (Bregs) in lupus nephritis patients. Scand J Rheumatol 2016;45:312–6. 26948375 10.3109/03009742.2015.1126346

[52] Knippenberg S , Peelen E, Smolders J et al Reduction in IL-10 producing B cells (Breg) in multiple sclerosis is accompanied by a reduced naive/memory Breg ratio during a relapse but not in remission. J Neuroimmunol 2011;239:80–6. 21940055 10.1016/j.jneuroim.2011.08.019

[53] Hu HT , Ai X, Lu M et al Characterization of intratumoral and circulating IL-10-producing B cells in gastric cancer. Exp Cell Res 2019;384:111652. 31574287 10.1016/j.yexcr.2019.111652

[54] Garaud S , Buisseret L, Solinas C et al Tumor infiltrating B-cells signal functional humoral immune responses in breast cancer. JCI Insight 2019;5:10.1172/jci.insight.129641 PMC6795287 31408436

[55] Horikawa M , Weimer ET, DiLillo DJ et al Regulatory B cell (B10 Cell) expansion during Listeria infection governs innate and cellular immune responses in mice. J Immunol 2013;190:1158–68. 23275601 10.4049/jimmunol.1201427 PMC3552111

[56] Lighaam LC , Unger P-PA, Vredevoogd DW et al In vitro-induced human IL-10(+) B cells Do not show a subset-defining marker signature and plastically co-express IL-10 with pro-inflammatory cytokines. Front Immunol 2018;9:1913. 30258433 10.3389/fimmu.2018.01913 PMC6143818

[57] Yang S-Y , Long J, Huang M-X et al Characterization of organ-specific regulatory B cells using single-cell RNA sequencing. Front Immunol 2021;12:711980. 34594327 10.3389/fimmu.2021.711980 PMC8476928

[58] Yanaba K , Bouaziz J-D, Haas KM et al A regulatory B cell subset with a unique CD1dhiCD5+ phenotype controls T cell-dependent inflammatory responses. Immunity 2008;28:639–50. 18482568 10.1016/j.immuni.2008.03.017

[59] Iwata Y , Matsushita T, Horikawa M et al Characterization of a rare IL-10-competent B-cell subset in humans that parallels mouse regulatory B10 cells. Blood 2011;117:530–41. 20962324 10.1182/blood-2010-07-294249 PMC3031478

[60] Matsushita T , Tedder TF. Identifying regulatory B cells (B10 cells) that produce IL-10 in mice. Methods Mol Biol 2011;677:99–111. 20941605 10.1007/978-1-60761-869-0_7

[61] Hasan MM , Thompson-Snipes LAnn, Klintmalm G et al CD24(hi)CD38(hi) and CD24(hi)CD27(+) human regulatory B cells display common and distinct functional characteristics. J Immunol 2019;203:2110–20. 31511354 10.4049/jimmunol.1900488

[62] Wu H , Su Z, Barnie PA. The role of B regulatory (B10) cells in inflammatory disorders and their potential as therapeutic targets. Int Immunopharmacol 2020;78:106111. 31881524 10.1016/j.intimp.2019.106111

[63] Berthelot J-M , Jamin C, Amrouche K et al Regulatory B cells play a key role in immune system balance. Joint Bone Spine 2013;80:18–22. 22858147 10.1016/j.jbspin.2012.04.010

[64] Yanaba K , Bouaziz JD, Matsushita T et al The development and function of regulatory B cells expressing IL-10 (B10 cells) requires antigen receptor diversity and TLR signals. J Immunol 2009;182:7459–72. 19494269 10.4049/jimmunol.0900270 PMC3733128

[65] Qian L , Bian G-R, Zhou Y et al Clinical significance of regulatory B cells in the peripheral blood of patients with oesophageal cancer. Cent Eur J Immunol 2015;40:263–5. 26557042 10.5114/ceji.2015.52840 PMC4637401

[66] Tao H , Lu L, Xia Y et al Antitumor effector B cells directly kill tumor cells via the Fas/FasL pathway and are regulated by IL-10. Eur J Immunol 2015;45:999–1009. 25545618 10.1002/eji.201444625 PMC4414939

[67] Das A , Ellis G, Pallant C et al IL-10-producing regulatory B cells in the pathogenesis of chronic hepatitis B virus infection. J Immunol 2012;189:3925–35. 22972930 10.4049/jimmunol.1103139 PMC3480715

[68] Gutiérrez C , Lopez-Abente J, Pérez-Fernández V et al Analysis of the dysregulation between regulatory B and T cells (Breg and Treg) in human immunodeficiency virus (HIV)-infected patients. PLoS One 2019;14:e0213744. 30917149 10.1371/journal.pone.0213744 PMC6436717

[69] Fillatreau S , Sweenie CH, McGeachy MJ et al B cells regulate autoimmunity by provision of IL-10. Nat Immunol 2002;3:944–50. 12244307 10.1038/ni833

[70] Ummarino D. Rheumatoid arthritis: defective IL-10-producing B(reg) cells. Nat Rev Rheumatol 2017;13:132. 10.1038/nrrheum.2017.10 28148914

[71] Wirz OF , Głobińska A, Ochsner U et al Comparison of regulatory B cells in asthma and allergic rhinitis. Allergy 2019;74:815–8. 30449036 10.1111/all.13672

[72] Choi JK , Egwuagu CE. Interleukin 35 regulatory B cells. J Mol Biol 2021;433:166607. 32755620 10.1016/j.jmb.2020.07.019 PMC7779660

[73] Turnis ME , Sawant DV, Szymczak-Workman AL et al Interleukin-35 limits anti-tumor immunity. Immunity 2016;44:316–29. 26872697 10.1016/j.immuni.2016.01.013 PMC4758699

[74] Dambuza IM , He C, Choi JK et al IL-12p35 induces expansion of IL-10 and IL-35-expressing regulatory B cells and ameliorates autoimmune disease. Nat Commun 2017;8:719. 28959012 10.1038/s41467-017-00838-4 PMC5620058

[75] Ye Z , Jiang Y, Sun D et al The plasma interleukin (IL)-35 level and frequency of circulating IL-35(+) regulatory b cells are decreased in a cohort of Chinese patients with new-onset systemic lupus erythematosus. Sci Rep 2019;9:13210. 31519970 10.1038/s41598-019-49748-z PMC6744462

[76] Wang S , Qin C. Interleukin 35 rescues regulatory B cell function, but the effect is dysregulated in ulcerative colitis. DNA Cell Biol 2017;36:413–21. 28398870 10.1089/dna.2016.3570

[77] Pylayeva-Gupta Y , Das S, Handler JS et al IL35-producing B cells promote the development of pancreatic neoplasia. Cancer Discov 2016;6:247–55. 26715643 10.1158/2159-8290.CD-15-0843 PMC5709038

[78] Senturk ZN , Akdag I, Deniz B, Sayi-Yazgan A. Pancreatic cancer: emerging field of regulatory B-cell-targeted immunotherapies. Front Immunol 2023;14:1152551. 37033931 10.3389/fimmu.2023.1152551 PMC10076755

[79] Huai G , Markmann JF, Deng S, Rickert CG. TGF-beta-secreting regulatory B cells: unsung players in immune regulation. Clin Transl Immunology 2021;10:e1270. 33815797 10.1002/cti2.1270 PMC8017464

[80] Bjarnadóttir K , Benkhoucha M, Merkler D et al B cell-derived transforming growth factor-β1 expression limits the induction phase of autoimmune neuroinflammation. Sci Rep 2016;6:34594–14. 27708418 10.1038/srep34594 PMC5052622

[81] Lee JH , Noh J, Noh G et al Allergen-specific transforming growth factor-beta-producing CD19+CD5+ regulatory B-cell (Br3) responses in human late eczematous allergic reactions to cow's milk. J Interferon Cytokine Res 2011;31:441–9. 21291325 10.1089/jir.2010.0020

[82] Cohen NR , Garg S, Brenner MB. Antigen presentation by CD1 lipids, T Cells, and NKT cells in microbial immunity. Adv Immunol 2009;102:1–94. 19477319 10.1016/S0065-2776(09)01201-2

[83] Brennan PJ , Brigl M, Brenner MB. Invariant natural killer T cells: an innate activation scheme linked to diverse effector functions. Nat Rev Immunol 2013;13:101–17. 23334244 10.1038/nri3369

[84] Tedder TF. B10 cells: a functionally defined regulatory B cell subset. J Immunol 2015;194:1395–401. 25663677 10.4049/jimmunol.1401329

[85] Bosma A , Abdel-Gadir A, Isenberg DA et al Lipid-antigen presentation by CD1d+ B cells is essential for the maintenance of invariant natural killer T cells. Immunity 2012;36:477–90. 22406267 10.1016/j.immuni.2012.02.008 PMC3391684

[86] Sun X , Zhang T, Li M et al Immunosuppressive B cells expressing PD-1/PD-L1 in solid tumors: a mini review. QJM 2022;115:507–12. 31250021 10.1093/qjmed/hcz162

[87] Zhang Y , Morgan R, Chen C et al Mammary-tumor-educated B cells acquire LAP/TGF-beta and PD-L1 expression and suppress anti-tumor immune responses. Int Immunol 2016;28:423–33. 26895637 10.1093/intimm/dxw007 PMC5006091

[88] Xiao X , Lao X-M, Chen M-M et al PD-1hi identifies a novel regulatory B-cell population in human hepatoma that promotes disease progression. Cancer Discov 2016;6:546–59. 26928313 10.1158/2159-8290.CD-15-1408

[89] Wang Z , Tan F. The blockade of PD-1/PD-L1 pathway promotes the apoptosis of CD19+CD25+ Bregs and suppresses the secretion of IL-10 in patients with allergic rhinitis. Scand J Immunol 2020;91:e12836. 31598989 10.1111/sji.12836

[90] Kaltenmeier C , Gawanbacht A, Beyer T et al CD4+ T cell-derived IL-21 and deprivation of CD40 signaling favor the in vivo development of granzyme B-expressing regulatory B cells in HIV patients. J Immunol 2015;194:3768–77. 25780036 10.4049/jimmunol.1402568

[91] Chesneau M , Michel L, Dugast E et al Tolerant kidney transplant patients produce B cells with regulatory properties. J Am Soc Nephrol 2015;26:2588–98. 25644114 10.1681/ASN.2014040404 PMC4587683

[92] Xu L , Liu X, Liu H et al Impairment of granzyme B-producing regulatory B cells correlates with exacerbated rheumatoid arthritis. Front Immunol 2017;8:768. 28713386 10.3389/fimmu.2017.00768 PMC5491972

[93] Kuchroo VK , Meyers JH, Umetsu DT, DeKruyff RH. TIM family of genes in immunity and tolerance. Adv Immunol 2006;91:227–49. 16938542 10.1016/S0065-2776(06)91006-2

[94] Kuchroo VK , Dardalhon V, Xiao S, Anderson AC. New roles for TIM family members in immune regulation. Nat Rev Immunol 2008;8:577–80. 18617884 10.1038/nri2366

[95] Umetsu SE , Lee W-L, McIntire JJ et al TIM-1 induces T cell activation and inhibits the development of peripheral tolerance. Nat Immunol 2005;6:447–54. 15793575 10.1038/ni1186

[96] Guo H , Shen Y, Kong Y-H et al The expression of Tim-1 and Tim-4 molecules in regulatory T cells in type 1 diabetes. Endocrine 2020;68:64–70. 31916216 10.1007/s12020-019-02173-8

[97] Degauque N , Mariat C, Kenny J et al Immunostimulatory Tim-1-specific antibody deprograms Tregs and prevents transplant tolerance in mice. J Clin Invest 2008;118:735–41. 18079964 10.1172/JCI32562 PMC2129234

[98] Xiao S , Brooks CR, Sobel RA, Kuchroo VK. Tim-1 is essential for induction and maintenance of IL-10 in regulatory B cells and their regulation of tissue inflammation. J Immunol 2015;194:1602–8. 25582854 10.4049/jimmunol.1402632 PMC4346345

[99] Xue H , Lin F, Tan H et al Overrepresentation of IL-10-expressing B cells suppresses cytotoxic CD4+ T cell activity in HBV-induced hepatocellular carcinoma. PLoS One 2016;11:e0154815. 27136203 10.1371/journal.pone.0154815 PMC4852942

[100] Liu Y , Chen Z, Qiu J et al Altered Tim-1 and IL-10 expression in regulatory B Cell Subsets in Type 1 Diabetes. Front Immunol 2021;12:773896. 35754999 10.3389/fimmu.2021.773896 PMC9231524

[101] Bod L , Kye Y-C, Shi J et al B-cell-specific checkpoint molecules that regulate anti-tumour immunity. Nature 2023;619:348–56. 37344597 10.1038/s41586-023-06231-0 PMC10795478

[102] Tian X , Zheng X, Tian D. B-cell immune checkpoint TIM-1: a potential target for tumour immunotherapy. Signal Transduct Target Ther 2023;8:389. 37857611 10.1038/s41392-023-01643-w PMC10587137

[103] Wang T , Mei Y, Li Z. Research progress on regulatory B cells in systemic lupus erythematosus. Biomed Res Int 2019;2019:7948687.(). 31240224 10.1155/2019/7948687 PMC6556307

[104] Yang X , Yang J, Chu Y et al T follicular helper cells and regulatory B cells dynamics in systemic lupus erythematosus. PLoS One 2014;9:e88441. 24551101 10.1371/journal.pone.0088441 PMC3925141

[105] Menon M , Blair PA, Isenberg DA, Mauri C. A regulatory feedback between plasmacytoid dendritic cells and regulatory B cells is aberrant in systemic lupus erythematosus. Immunity 2016;44:683–97. 26968426 10.1016/j.immuni.2016.02.012 PMC4803914

[106] Dai YC , Zhong J, Xu JF. Regulatory B cells in infectious disease (Review). Mol Med Rep 2017;16:3–10. 28534949 10.3892/mmr.2017.6605 PMC5482109

[107] Lopez-Abente J , Prieto-Sanchez A, Munoz-Fernandez MA et al Human immunodeficiency virus type-1 induces a regulatory B cell-like phenotype in vitro. Cell Mol Immunol 2018;15:917–33. 28713164 10.1038/cmi.2017.48 PMC6207566

[108] Zhivaki D , Lemoine S, Lim A et al Respiratory syncytial virus infects regulatory B cells in human neonates via chemokine receptor CX3CR1 and promotes lung disease severity. Immunity 2017;46:301–14. 28228284 10.1016/j.immuni.2017.01.010 PMC7128247

[109] Cervantes-Díaz R , Sosa-Hernández VA, Romero-Ramírez S et al Circulating B10 regulatory cells are decreased in severe and critical COVID-19. J Leukoc Biol 2022;112:333–7. 35199888 10.1002/JLB.5COVCRA0721-387RR PMC9088486

[110] Zhao Y , Shen M, Feng Y et al Regulatory B cells induced by pancreatic cancer cell-derived interleukin-18 promote immune tolerance via the PD-1/PD-L1 pathway. Oncotarget 2018;9:14803–14. 29599908 10.18632/oncotarget.22976 PMC5871079

[111] Oka A , Ishihara S, Mishima Y et al Role of regulatory B cells in chronic intestinal inflammation: association with pathogenesis of Crohn's disease. Inflamm Bowel Dis 2014;20:315–28. 24390063 10.1097/01.MIB.0000437983.14544.d5

[112] van der Vlugt LEPM , Zinsou JF, Ozir-Fazalalikhan A et al Interleukin 10 (IL-10)–producing CD1dhi regulatory B cells from schistosoma haematobium–infected individuals induce IL-10–positive T cells and suppress effector T-cell cytokines. J Infect Dis 2014;210:1207–16. 24795476 10.1093/infdis/jiu257

[113] Hetta HF , Mekky MA, Zahran AM et al Regulatory B cells and their cytokine profile in HCV-related hepatocellular carcinoma: association with regulatory T cells and disease progression. Vaccines (Basel) 2020;8:380. 32664587 10.3390/vaccines8030380 PMC7565874

[114] Shao Y , Lo CM, Ling CC et al Regulatory B cells accelerate hepatocellular carcinoma progression via CD40/CD154 signaling pathway. Cancer Lett 2014;355:264–72. 25301451 10.1016/j.canlet.2014.09.026

[115] Lv Y , Wang H, Liu Z. The role of regulatory B cells in patients with acute myeloid leukemia. Med Sci Monit 2019;25:3026–31. 31017878 10.12659/MSM.915556 PMC6496973

[116] Guan H , Lan Y, Wan Y et al PD-L1 mediated the differentiation of tumor-infiltrating CD19+ B lymphocytes and T cells in Invasive breast cancer. OncoImmunology 2016;5:e1075112. 27057444 10.1080/2162402X.2015.1075112 PMC4801469

[117] Ma Q-Y , Chen J, Zhao J. Follicular cytotoxic CD8 T cells present high cytokine expression, and are more susceptible to Breg-mediated suppression in non-small cell lung cancer. Immunol Res 2020;68:54–62. 32128664 10.1007/s12026-020-09120-0

[118] Siewe B , Stapleton JT, Martinson J et al Regulatory B cell frequency correlates with markers of HIV disease progression and attenuates anti‐HIV CD8+ T cell function in vitro. J Leukoc Biol 2013;93:811–8. 23434518 10.1189/jlb.0912436 PMC3629440

[119] Wang Y , Qin Y, Wang X et al Decrease in the proportion of CD24(hi) CD38(hi) B cells and impairment of their regulatory capacity in type 1 diabetes patients. Clin Exp Immunol 2020;200:22–32. 31849037 10.1111/cei.13408 PMC7066388

[120] Flores-Borja F , Bosma A, Ng D et al CD19+ CD24hiCD38hi B cells maintain regulatory T cells while limiting TH1 and TH17 differentiation. Sci Transl Med 2013;5:173ra23. 10.1126/scitranslmed.3005407 23427243

[121] Daien CI , Gailhac S, Mura T et al Regulatory B10 cells are decreased in patients with rheumatoid arthritis and are inversely correlated with disease activity. Arthritis Rheumatol 2014;66:2037–46. 24729478 10.1002/art.38666

[122] Wang X , Li J, Lu C et al IL-10-producing B cells in differentiated thyroid cancer suppress the effector function of T cells but improve their survival upon activation. Exp Cell Res 2019;376:192–7. 30711567 10.1016/j.yexcr.2019.01.021

[123] Jeske SS , Brand M, Ziebart A et al Adenosine-producing regulatory B cells in head and neck cancer. Cancer Immunol Immunother 2020;69:1205–16. 32146518 10.1007/s00262-020-02535-6 PMC7303082

[124] Wang X , Zhu Y, Zhang M et al Ulcerative colitis is characterized by a decrease in regulatory B cells. J Crohns Colitis 2016;10:1212–23. 26980839 10.1093/ecco-jcc/jjw074

[125] Wiest M , Upchurch K, Hasan MM et al Phenotypic and functional alterations of regulatory B cell subsets in adult allergic asthma patients. Clin Exp Allergy 2019;49:1214–24. 31132180 10.1111/cea.13439

[126] Mao Y , Wang Y, Dong L et al Circulating exosomes from esophageal squamous cell carcinoma mediate the generation of B10 and PD‐1high Breg cells. Cancer Sci 2019;110:2700–10. 31276257 10.1111/cas.14122 PMC6726703

[127] Chen Z , Zhu Y, Du R et al Role of regulatory B cells in the progression of cervical cancer. Mediators Inflamm 2019;2019:6519427. 31316301 10.1155/2019/6519427 PMC6604409

[128] Ye L , Zhang Q, Cheng Y et al Tumor-derived exosomal HMGB1 fosters hepatocellular carcinoma immune evasion by promoting TIM-1+ regulatory B cell expansion. J Immunother Cancer 2018;6:145. 30526680 10.1186/s40425-018-0451-6 PMC6288912

[129] Zacca ER , Onofrio LI, Acosta CDV et al PD-L1+ regulatory B cells are significantly decreased in rheumatoid arthritis patients and increase after successful treatment. Front Immunol 2018;9:2241. 30327652 10.3389/fimmu.2018.02241 PMC6174216

[130] Liu J , Zhan W, Kim CJ et al IL-10-producing B cells are induced early in HIV-1 infection and suppress HIV-1-specific T cell responses. PloS One 2014;9:e89236. 24586620 10.1371/journal.pone.0089236 PMC3931714

[131] Shang J , Zha H, Sun Y. Phenotypes, functions, and clinical relevance of regulatory B cells in cancer. Front Immunol 2020;11:582657. 33193391 10.3389/fimmu.2020.582657 PMC7649814

[132] Zhang L , Tai Y-T, Ho M et al Regulatory B cell-myeloma cell interaction confers immunosuppression and promotes their survival in the bone marrow milieu. Blood Cancer J 2017;7:e547. 28338671 10.1038/bcj.2017.24 PMC5380908

[133] Shen M , Wang J, Yu W et al A novel MDSC-induced PD-1(-)PD-L1(+) B-cell subset in breast tumor microenvironment possesses immuno-suppressive properties. Oncoimmunology 2018;7:e1413520. 29632731 10.1080/2162402X.2017.1413520 PMC5889195

[134] Wortel CM , Heidt S. Regulatory B cells: phenotype, function and role in transplantation. Transpl Immunol 2017;41:1–9. 28257995 10.1016/j.trim.2017.02.004

[135] Yeung MY , Ding Q, Brooks CR et al TIM-1 signaling is required for maintenance and induction of regulatory B cells. Am J Transplant 2015;15:942–53. 25645598 10.1111/ajt.13087 PMC4530122

[136] Deng S , Moore DJ, Huang X et al Cutting edge: transplant tolerance induced by anti-CD45RB requires B lymphocytes. J Immunol 2007;178:6028–32. 17475825 10.4049/jimmunol.178.10.6028

[137] Lee KM , Stott RT, Zhao G et al TGF-beta-producing regulatory B cells induce regulatory T cells and promote transplantation tolerance. Eur J Immunol 2014;44:1728–36. 24700192 10.1002/eji.201344062 PMC4048633

[138] Asare A , Kanaparthi S, Lim N et al B cell receptor genes associated with tolerance identify a cohort of immunosuppressed patients with improved renal allograft graft function. Am J Transplant 2017;17:2627–39. 28371372 10.1111/ajt.14283 PMC5620117

[139] Cherukuri A , Rothstein DM, Clark B et al Immunologic human renal allograft injury associates with an altered IL-10/TNF-alpha expression ratio in regulatory B cells. J Am Soc Nephrol 2014;25:1575–85. 24610932 10.1681/ASN.2013080837 PMC4073434

[140] Cherukuri A , Salama AD, Mehta R et al Transitional B cell cytokines predict renal allograft outcomes. Sci Transl Med 2021;13:10.1126/scitranslmed.abe4929 33627487

[141] Yarchoan M , Mohan AA, Dennison L et al MEK inhibition suppresses B regulatory cells and augments anti-tumor immunity. PLoS One 2019;14:e0224600. 31671149 10.1371/journal.pone.0224600 PMC6822709

[142] Bodogai M , Lee Chang C, Wejksza K et al Anti-CD20 antibody promotes cancer escape via enrichment of tumor-evoked regulatory B cells expressing low levels of CD20 and CD137L. Cancer Res 2013;73:2127–38. 23365136 10.1158/0008-5472.CAN-12-4184 PMC3618504

[143] Selitsky SR , Mose LE, Smith CC et al Prognostic value of B cells in cutaneous melanoma. Genome Med 2019;11:36. 31138334 10.1186/s13073-019-0647-5 PMC6540526

[144] De Marco RC , Monzo HJ, Ojala PM. CAR T cell therapy: a versatile living drug. Int J Mol Sci 2023;24:2–22. 10.3390/ijms24076300 PMC10094630 37047272

[145] Mauri C , Menon M. Human regulatory B cells in health and disease: therapeutic potential. J Clin Invest 2017;127:772–9. 28248202 10.1172/JCI85113 PMC5330739

